# Integrative Role of *14-3-3ε* in Sleep Regulation

**DOI:** 10.3390/ijms22189748

**Published:** 2021-09-09

**Authors:** Yu Wei, Juan Du, Zhangwu Zhao

**Affiliations:** Department of Entomology and MOA Key Lab of Pest Monitoring and Green Management, College of Plant Protection, China Agricultural University, Beijing 100193, China; weiyusmiling@163.com

**Keywords:** sleep, clock, *14-3-3ε*, *PDF*, *Drosophila*

## Abstract

Sleep is a crucial factor for health and survival in all animals. In this study, we found by proteomic analysis that some cancer related proteins were impacted by the circadian clock. The *14-3-3ε* protein, expression of which is activated by the circadian transcription factor *Clock*, regulates adult sleep of Drosophila independent of circadian rhythm. Detailed analysis of the sleep regulatory mechanism shows that *14-3-3ε* directly targets the Ultrabithorax (*Ubx*) gene to activate transcription of the pigment dispersing factor (*PDF*). The dopamine receptor (*Dop1R1*) and the octopamine receptor (*Oamb*), are also involved in the *14-3-3ε* pathway, which in *14-3-3ε* mutant flies causes increases in the *dopR1* and *OAMB*, while downregulation of the *DopR1* and *Oamb* can restore the sleep phenotype caused by the *14-3-3ε* mutation. In conclusion, *14-3-3ε* is necessary for sleep regulation in *Drosophila*.

## 1. Introduction

Sleep is very important for the health and survival of animals, and it is regulated mainly by the circadian rhythm and homeostasis [1,2,3]. Sleep has been extensively studied in the model animal *Drosophila melanogaster*, which is detected by measuring the activity through a *Drosophila* activity monitoring (DAM) system [4]. Fly immobility for 5 min or longer is defined as sleep [5,6].

In *Drosophila*, approximately 150 clock neurons in the central nervous system are involved in circadian rhythms, mainly including LNvs (ventral lateral neurons), LNds (dorsal lateral neurons) and DN1s (dorsal neurons) to form a feedback loop to control sleep-activity of Drosophila. The PDF-positive l-LNvs and s-LNvs (M cells) are known as arousal neurons. Loss of PDF neurons or PDF itself increases the amount of daytime sleep, while the CRY-positive LNds and the 5th s-LNv (E cells) control the amount of nighttime sleep; activation of the E cells causes sleep loss [7,8,9,10]. In addition, PDF neurons also modulate the phase of E cell oscillations [8,11]. PDF containing s-LNv dorsal projections exhibit a clock-controlled structural plasticity [12], in which some genes and microRNAs such as the adipokinetic hormone (AKH) [13] and microRNA-263b [14] involved in s-LNvs axonal fasciculation have been shown to impact circadian behavior. 

The *14-3-3* family is highly conserved in protein sequence and function from yeast to mammals. They are involved in some biological processes such as cell proliferation, and apoptosis [15,16]. There are seven *14-3-3* members in vertebrates separately named ζ, δ, β, ε, γ, η, and θ according to their amino acid sequences. In *Drosophila*, there are two paralogs of *14-3-3* protein, ζ and ε, participating in both the Hippo pathway and the Ras/MAPK pathway [17,18,19]. Biochemistry data have shown that the isolated η chain of *14-3-3* protein from bovine brain can activate tyrosine hydroxylase and tryptophan hydroxylase in the presence of Ca^2+^/calmodulin-dependent protein kinase type II [20]. Inhibition of the *14-3-3* family of proteins results in functional reduction of glutamatergic synapses [21]. However, whether *14-3-3* is related to sleep is still unclear. 

The *Drosophila* CLOCK (CLK) is one of the most important core oscillation proteins in the biological clock for controlling daily circadian rhythms and sleep, and its deficiency may result in a disorder of circadian rhythms and abnormal sleep. Therefore, we used the *Clock*-deficient mutant (*Clk^Jrk^*) and the same background of wild-type (*w^1118^*) control flies to screen and identify the downstream circadian-related genes, in which the *14-3-3ε* is found to be a sleep-regulating factor related to *Clock.* Thus, we focused on its mechanism of sleep regulation.

## 2. Materials and Methods

### 2.1. Fly Stocks

The following stocks were used in this study: *Amph^26^*, *Pss^HP31723^*, *14-3-3ε^EP3578^*, *14-3-3ε^j2b10^/+*, *Ubx*-gal4/tm6b, UAS-mRFP, UAS-*14-3-3ε^RNAi^*, *pdf*-gal4, *14-3-3ε^G00082^*, *Dop1R1^KO^* and *Oamb^MI11478^*. *Amph^26^* (BS6498), *Pss^HP31723^* (BS22115), *14-3-3ε^EP3578^* (BS17142), *14-3-3ε^j2b10^/+* (BS12142), *pdf*-gal4 (BS41286), *Oamb^MI11478^* (BS56423), and *14-3-3ε^G00082^* (BS51385) were purchased from the Bloomington Drosophila Stock Center. UAS-*14-3-3ε^RNAi^* (v15884) was purchased from the Vienna Drosophila Resource Center. *Dop1R1^KO^* was a gift from Dr. Yi Rao’s lab [22]. *14-3-3ε^EP3578^* and *14-3-3ε^j2b10^/+* mutants were derived by insertional mutagenesis using the different P-element constructs. They were backcrossed by *w^1118^* for six generations. 

All flies were reared at 25 °C and 65% relative humidity with standard corn flour/yeast/agar food supplemented with CaCl_2_ in a 12 h light/12 h dark cycle.

### 2.2. Behavioral Assays

Three to five day-old male adults were housed in monitor tubes (5[W] × 65[L] mm) with fly food. Experiments were performed in a Climate box at 25 ± 1 °C with 50% relative humidity. Light was turned on at ZT0 (06:30) and off at ZT12 (18:30). The activity data were recorded using the *Drosophila* Activity Monitoring System (Tri-kinetics, Waltham, MA, USA). The protocol and data analysis are described in Chen et al. (2013) [23]. 

### 2.3. Immunofluorescence

The flies were immobilized in 4% paraformaldehyde for 12 h at 4˚C and were then dissected in phosphate-buffered saline (PBS). The brains were blocked in blocking buffer (10% Normal Goat Serum diluted with 2% PBST) at RT for 2 h. The tissue was incubated in primary antibody for 24 h at 4 °C before being incubated with secondary antibodies overnight at 4 °C. The primary antibodies were as follows: mouse anti-PDF (DSHB UAS Cat# C7 monoclonal antibody; 1:400), rabbit anti-GFP (Invitrogen UAS Cat# PA1-980A polyclonal antibody; 1:400), and mouse anti-RFP (Abclonal China Cat# AE020 monoclonal antibody; 1:50). Fluorescent secondary antibodies conjugated to Goat anti-Rabbit FITC (Abclonal China Cat# AS011; 1:100) and Goat anti-Mouse TRITC (Abclonal China Cat# AS026; 1:100). The immunofluorescence assay was carried out on a Leica system (Leica SP8, Wetzlar, Germany).

### 2.4. Total RNA Isolation, cDNA Synthesis, and Quantitative Real-Time PCR

Total RNA was isolated from heads of five to seven-day-old flies using RNAiso plus (TaKaRa Japan Cat# 9109). Each sample contained 30 individual flies with three biological repeats, which were reversely transcribed and measured by real-time PCR, respectively. There were three technical repeats in each biological repeat in the real-time PCR experiment. The total RNA quality was checked by Agilent Bioanalyser. A total of 1 μg RNA was added in each reverse transcription system. The RNA was reversely transcribed with a PrimeScrip^TM^ RT reagent Kit with gDNA Eraser (TaKaRa Japan Cat# RR047A). A total of 1μL cDNA was added in each real-time PCR reaction system. SYBR Green method was used for Real-time PCR with SuperReal PreMix Plus kit (TIANGEN China Cat# FP205-02). The *PDF* gene real-time PCR program: holding stage 95 °C 10mins; cycling stage 95 °C 15 s, 57 °C 25 s, 68 °C 35 s, 40 cycles; melt curve stage 95 °C 15 s, 60 °C 1 min, temperature increment +0.3 °C, 95 °C 15 s. The *Ubx* gene real-time PCR program: holding stage 95 °C 10 min; cycling stage 95 °C 15 s, 60 °C 20 s, 72 °C 30 s, 40cycles; melt curve stage 95 °C 15 s, 60 °C 1 min, temperature increment +0.3 °C, 95 °C 15 s. *RP49* (also named as *RpL32*) was regarded as reference gene. *w^1118^* control was used for normalization. The △△CT method was used for quantification. The real-time PCR data analysis is described in Livak et al. (2001) [24]. The real-time PCR assay was carried out on an Applied Biosystem Step One Real-Time PCR system (Applied Biosystem, Foster, CA, USA). The primers were designed by Beacon Designer 8. The sequences of the primers are shown in Table S4. 

### 2.5. Western Blot Analysis and Co-Immunoprecipitation

*D. melanogaster* heads were collected and lysed with strong lysis buffer (CWBIO China Cat# CW2333) and protease inhibitor (CWBIO China Cat# CW2200). Whole tissue lysates were subjected to SDS-PAGE and immunoblotting as described (REF). The molecular weights of *14-3-3ε*, Ubx and β-tubulin protein are 30KDa, 40KDa and 50KDa, respectively. The used primary antibodies were as follows: guinea pig anti-*14-3-3ε* (1:1000, from Aurelio A. Teleman as gift), mouse anti-*Ubx* (DSHB UAS Cat# FP3.38 monoclonal antibody; 1:50), and mouse anti-*β-tubulin* (Abclonal China Cat# AC010 monoclonal antibody; 1:1000). The used secondary antibodies were as follows: HRP Goat anti-Guinea Pig IgG (Abclonal China Cat# AS025; 1:2000), HRP Goat anti-Mouse IgG (Abclonal China Cat# AS003; 1:2000). Co-immunoprecipitation was conducted as previously described [25]. 

### 2.6. Chromatin Immunoprecipitation Assay

Chromatin immunoprecipitation was conducted as previously described [26]. *w^1118^* strain fly heads were collected and fixed by shaking in 1% formaldehyde for 10 min at RT, and cross-linking reactions were stopped by adding glycine at a final concentration of 125 mM at RT for 5 min. The cross-linked chromatin was cut by sonication to approximately 200–500 bp fragments. A 120 μL sample of protein was used for immunoprecipitation, and 10 μL was maintained as the input DNA. The chromatin immunoprecipitation reaction was performed with 20 μL of antibody (mouse anti-Ubx). Immunoprecipitated DNA was quantified by real-time PCR. The ChIP-qPCR data were normalized by the input DNA, and the results were presented as the enrichment fold DNA. The sequences of the primers are shown in Table S4. Each experiment was independently performed three times.

### 2.7. Calculation of Axonal Cross

Axonal cross was used to quantify the morphology complexity. The data analysis is described in Fernández et al. (2008) [12]. Six evenly spaced (10 μm) concentric rings centered at the point where the first dorsal ramification opens up were drawn on each brain hemisphere. The number of intersections of each projection with a particular ring were counted. The total number of intersections were compared using nonparametric statistical methods.

### 2.8. Proteomic Screen and RNA-Seq 

Total proteins from whole heads in both wild-type (*w^1118^*) and *Clk^Jrk^* mutant flies were sampled at ZT2, ZT8, ZT14 and ZT20 and analyzed using the iTRAQ-MS method. Ingenuity Pathway Analysis (IPA) was used for protein screening. For the detailed methods of experiment and data analysis of proteomic screen, refer to our previous publication [27]. The total RNA extracted from Drosophila heads of homozygote *14-3-3ε^EP3578^* and *w^1118^* at ZT2 and ZT14 was used for RNA-seq. The RNA-seq was completed by Beijing Biomics Biotech Co. Ltd. (Beijing, China). Each sample contained 70 individual flies.

### 2.9. Statistical Analysis

Statistical analysis was performed with SPSS statistics 18.0. *p* values were obtained with One-way ANOVA, Two-way ANOVA and unpaired Student’s *t*-test and were considered to indicate significance; n.s. no significant difference, * *p* < 0.05, ** *p* < 0.01, *** *p* < 0.001, and **** *p* < 0.0001.

## 3. Results

### 3.1. 14-3-3ε Regulates Sleep Independent of the Circadian Rhythm

**Figure 1:**
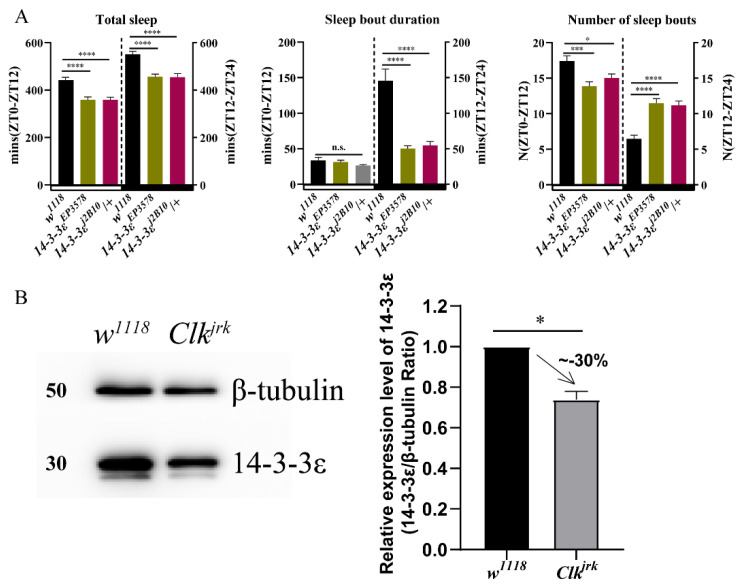
Sleep and the circadian locomotor rhythm in *14-3-3ε* mutants: (**A**), sleep pattern of *14-3-3ε* mutants. (**B**), 14-3-3ε protein expression level in *Clock* mutant (*Clk^Jrk^*) and *w^1118^* control flies by Western blotting. Bar graphs are presented as mean ± SEM. Statistical differences were measured using unpaired Student’s *t*-test; n.s. indicates no significant difference, * *p* < 0.05, *** *p* < 0.001, **** *p* < 0.0001. Each experiment was conducted in triplicate.

In order to identify potential circadian regulators, we conducted a proteomic screen for oscillating proteins that are differentially expressed in the *Drosophila* head between the wild-type *w^1118^* and *Clock*-deficient mutant *Clk^Jrk^* [27]. Surprisingly, we found that some cancer-related proteins were controlled by *Clock*, in which some genes were selected from non-phosphorylated and phosphorylated differential proteins between *w^1118^* and *Clk^Jrk^*. Behavioral analysis of the circadian rhythm and sleep from the mutants of some candidate genes showed that they had anomalous sleep phenotypes (Figure S1), in which we found that the *14-3-3ε* regulates sleep independent of the circadian rhythm, with a normal rhythmic percentage when compared to that of control (Figure 1A, Table S1). It is significantly decreased in the *Clk^Jrk^* mutant detected by the proteomic screen, further verified by using Western blotting (*p* = 0.023) (Figure 1B). Thus, we focused on its mechanism of sleep regulation in this study. 

### 3.2. 14-3-3ε Regulates Sleep Factor PDF

**Figure 2:**
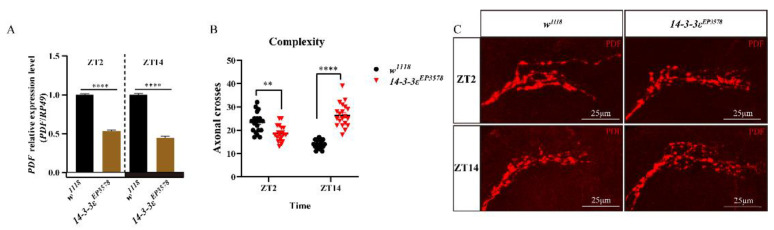
Effects of the *14-3-3ε* mutant on *PDF* expression and sLNv dorsal projections: (**A**), *PDF* mRNA expression level in *14-3-3ε* mutant (*14-3-3ε^EP3578^*) and *w^1118^* control flies at ZT2 and ZT14 by real-time PCR. (**B**), sLNv dorsal termini axonal cross in *14-3-3ε* mutant (*14-3-3ε^EP3578^*) and *w^1118^* control flies at ZT2 and ZT14. (**C**), sLNv dorsal projections in *14-3-3ε* mutant (*14-3-3ε^EP3578^*) and *w^1118^* control flies at ZT2 and ZT14 by immunofluorescence using PDF antibody (red). The scale bar indicates 25 μm. Bar graphs are presented as mean ± SEM. A, statistical differences were measured using unpaired Student’s *t*-test. (**B**), statistical differences were measured using Two-way ANOVA and Tukey’s multiple comparison test; ** *p* < 0.01, **** *p* < 0.0001. Each experiment was conducted in triplicate.

**Figure 3:**
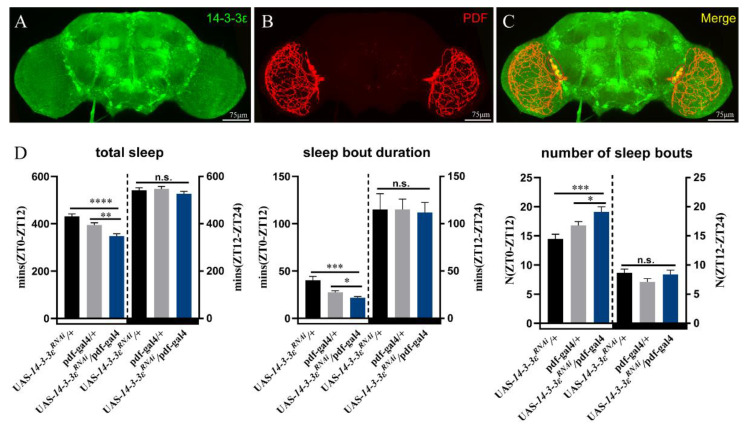
14-3-3ε function in the PDF neuron for sleep regulation: (**A**–**C**), expression pattern of 14-3-3ε in the adult fly brain by immunofluorescence. 14-3-3ε protein trap strain (*14-3-3ε^G00082^*, BS51385) fused with GFP (14-3-3ε-GFP), rabbit anti-GFP (14-3-3ε, 1:400, green), and mouse anti-PDF (PDF, 1:200, red). (**D**), sleep pattern with 14-3-3ε downregulation in PDF neuron. The scale bar indicates 75 μm. Statistical differences were measured using One-way ANOVA and Tukey’s multiple comparison test and unpaired Student’s *t*-test; n.s. indicates no significant difference, * *p* < 0.05, ** *p* < 0.01, *** *p* < 0.001, **** *p* < 0.0001. Each experiment was conducted in triplicate.

The pigment dispersing factor (PDF), expressed in the LNvs of clock neurons, is a regulatory factor of sleep in Drosophila. We found that the PDF transcription level decreased in the *14-3-3ε* mutant by transcriptomic analysis (Table S2), which was further verified by the real-time PCR, with significant decreases of 47% (*p* < 0.0001) at ZT2 and 55% (*p* < 0.0001) at ZT14 in the *14-3-3ε* mutant flies (*14-3-3ε^EP3578^*) compared to those in control flies (Figure 2A). Then, we detected the morphology of the PDF-containing sLNv dorsal projections in *14-3-3ε^EP3578^* and *w^1118^* flies, by which PDF signal transmits to the central complexes. The results showed that the morphology of dorsal projections changed greatly, in which the sLNv dorsal termini axonal cross at ZT2 was significantly decreased by 20% (*p* = 0.0011, *n* = 20) compared to that of the control, but it was significantly increased by 89% (*p* < 0.0001, *n* = 20) at ZT14 (Figure 2B,C). Furthermore, we used the *14-3-3ε* protein trap fly line (*14-3-3ε^G00082^*) fusing *14-3-3ε* with GFP (14-3-3ε-GFP) to co-locate the *14-3-3ε* and PDF (green for 14-3-3ε by rabbit anti-GFP and red for PDF by mouse anti-PDF). The results showed that both *14-3-3ε* and PDF co-expressed in the sLNvs (Figure 3A–C). 

Because *14-3-3ε* is expressed in the PDF neurons, we specifically downregulated its expression with an RNAi driven by *pdf-*gal4. The results showed that downregulation of *14-3-3ε* recapitulated the sleep phenotype caused by the *14-3-3ε* mutant, with significant decreases in the total sleep at daytime compared to that of controls (*p* < 0.0001 and *p* = 0.003, respectively) (Figure 3D). These results indicate that *14-3-3ε* regulates sleep via PDF in the PDF neurons.

### 3.3. 14-3-3ε Directly Acts on Ubx to Regulate PDF Transcription

**Figure 4:**
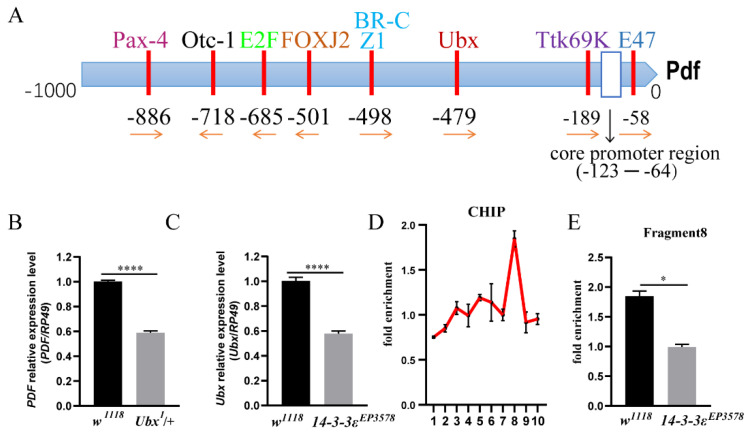
Ubx is a transcription factor of PDF: (**A**), Pdf transcription factor prediction. (**B**), *PDF* mRNA expression level in *Ubx* mutant (*Ubx^1^*/+) and *w^1118^* control flies by real-time PCR. (**C**), *Ubx* mRNA expression level in *14-3-3ε* mutant (*14-3-3ε^EP3578^*) and *w^1118^* control flies by real-time PCR. (**D**), chromatin immunoprecipitation with Ubx antibody and *w^1118^* adult heads as the sample. X-axis shows fragment numbers. (**E**), chromatin immunoprecipitation used the Ubx antibody, the *14-3-3ε* mutant (*14-3-3ε^EP3578^*), and *w^1118^* control adult heads as samples. Fragment 8 was tested by CHIP-qPCR. Bar graphs are presented as mean ± SEM. Statistical differences were measured using unpaired Student’s *t*-test; n.s. indicates no significant difference, * *p* < 0.05, **** *p* < 0.0001. Each experiment was conducted in triplicate.

To further determine how *14-3-3ε* regulates PDF, we predicted the transcription factors in the promotor of *PDF* using the website http://gene-regulation.com/index2.html (accessed on 1 October 2020), in which the *Ultrabithorax* (*Ubx*) is one of the transcription factors (Figure 4A). When *Ubx* was downregulated in the *Ubx* heterozygous mutant (*Ubx^1^*/+), the *pdf* mRNA level significantly decreased by 41% compared to that in the control (*p* < 0.0001) (Figure 4B). From the transcriptomic data (Table S2), the *Ubx* level decreased in the *14-3-3ε*-deficient mutant, which was further verified by real-time PCR (*p* < 0.0001) (Figure 4C). 

In order to determine the binding sites of *Ubx*, we designed 10 pairs of PCR primers spanning 2 kb upstream of the translational start sites of PDF (each fragment of which was around 200 bp) for analysis of DNA from ChIP (chromatin immunoprecipitation) with anti-Ubx. The results showed that there was an active peak in fragment 8 (Figure 4D), and the enrichment of this fragment was significantly decreased in the *14-3-3ε* mutant (0.99 vs. 1.85 times, *p* = 0.013) (Figure 4E). 

**Figure 5:**
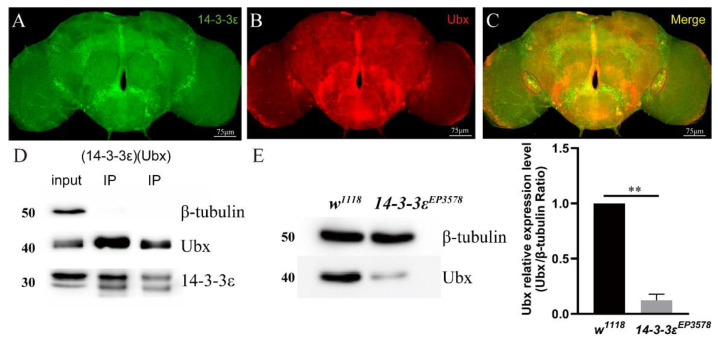
14-3-3ε interacts with Ubx: (**A**–**C**), immunofluorescence colocalization between 14-3-3ε and Ubx with mouse anti-RFP (Ubx, 1:100, red) and rabbit anti-GFP (14-3-3ε, 1:200, green) in UAS-mRFP/+; Ubx-gal4/+, 14-3-3ε-GFP/+ adult fly brain. (**D**), co-immunoprecipitation between 14-3-3ε and Ubx. First well, input. Second well, anti-14-3-3ε antibody for IP. Third well, anti-Ubx antibody for IP. *w^1118^* adult fly heads were used as the sample. (**E**), Ubx protein expression level in *14-3-3ε^EP3578^* and *w^1118^* by Western blotting. The scale bar (right bottom white line) indicates 75 μm. Bar graphs are presented as mean ± SEM. Statistical differences were measured using unpaired Student’s *t*-test; n.s. indicates no significant difference, ** *p* < 0.01. Each experiment was conducted in triplicate.

To gain more relationship between *14-3-3ε* and *Ubx*, we co-localized Ubx and *14-3-3ε* by using the *14-3-3ε*-GFP/+; *Ubx*-gal4/+ fly lines. The brains were stained with immunofluorescence using anti-GFP and anti-Ubx antibodies. The results showed that *14-3-3ε* and Ubx were merged together in PDF neurons (Figure 5A–C). Then, we employed co-immunoprecipitation experiments using anti-Ubx antibody and anti-14-3-3ε antibody, and results revealed that 14-3-3ε was able to directly combine with Ubx (Figure 5D). Furthermore, we quantified the Ubx protein in *14-3-3^EP3578^* by Western blotting. The evidence showed that Ubx significantly decreased by 88% when compared to control (*p* = 0.004) (Figure 5E). All these data indicate that *Ubx* is a direct target of *14-3-3ε*, which regulates PDF through activating the fragment 8 of *PDF.*

### 3.4. 14-3-3ε Regulates Sleep by Impacting Neurotransmitters 

**Figure 6:**
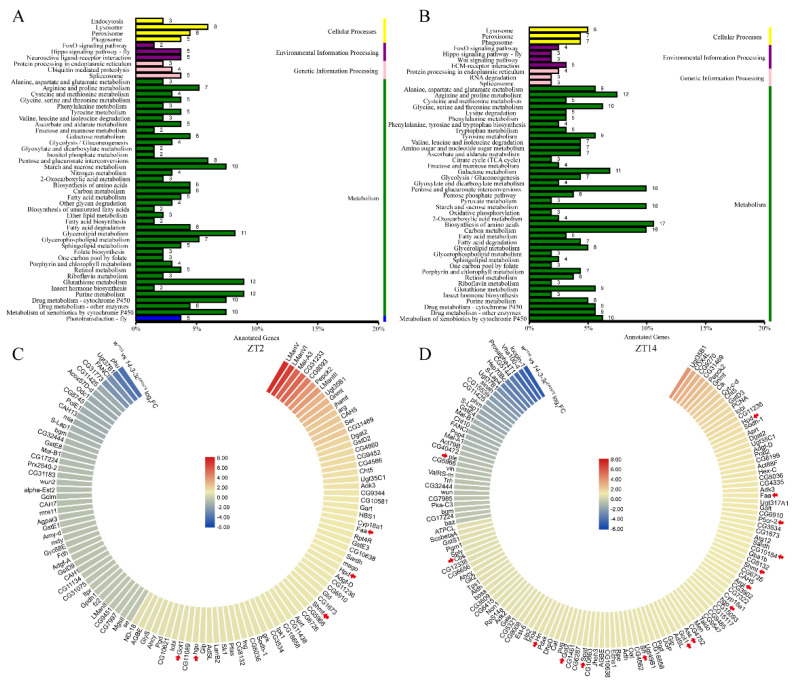
Neurotransmitter metabolism is changed when 14-3-3ε gene is mutated: (**A**,**B**), KEGG enrichment analysis of different genes in *14-3-3ε* mutant (*14-3-3ε^EP3578^*) transcriptome at ZT2 and ZT14. (**C**,**D**), the heatmaps of different genes relevant to metabolism from the transcriptome at ZT2 and ZT14. Red arrow displays differences for neurotransmitter metabolism genes.

To identify the molecular mechanism of *14-3-3ε* on sleep regulation, we performed RNA-seq of the head tissue at ZT2 and ZT14 in both the *14-3-3ε* deficient mutant (*14-3-3ε^EP3578^*) and *w^1118^* control flies (Table S2). The results showed that a number of differentially expressed genes between the mutant and control flies were related to metabolism, including glucometabolism, lipid metabolism, and amino acid metabolism (Figure 6A,B). These were classified into categories in which some of the genes are involved in the tyrosine metabolic process, the amino acid biosynthetic process of the glutamine family, and the amino acid metabolic process of the serine family (arrows in Figure 6C,D). Tyrosine, glutamate, and serine are important precursors for the synthesis of neurotransmitters. Most of them were upregulated at both ZT2 and ZT14 in *14-3-3ε^EP3578^* flies (Table S3). Specially, the differential genes between *w1118* and *14-3-3ε* mutant flies in tyrosine metabolism were involved in dopamine and octopamine synthesis process, in which *14-3-3ε* inhibits the production of dopamine and octopamine.

**Figure 7:**
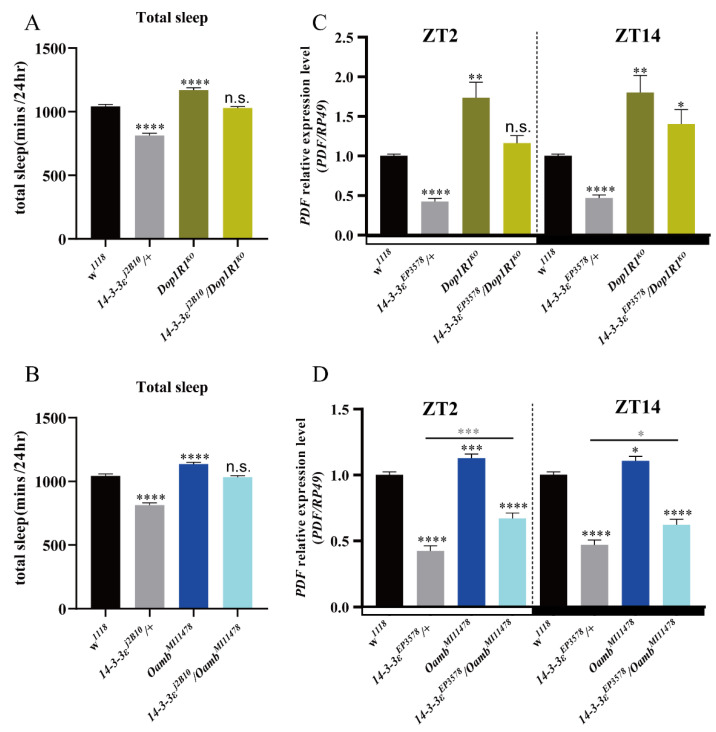
Neurotransmitter mutants rescue sleep loss induced by 14-3-3ε: (**A**), total sleep of *14-3-3ε* mutant as a double mutant with *Dop1R1*. (**B**), total sleep of *14-3-3ε* mutant as a double mutant with *Oamb*. (**C**), *PDF* mRNA expression level of *14-3-3ε* mutant as a double mutant with *Dop1R1* by real-time PCR. (**D**), *PDF* mRNA expression level of *14-3-3ε* mutant as a double mutant with *Oamb* by real-time PCR. Black star, compared with *w^1118^* control; gray star, compared with *14-3-3ε^EP3578^/+*. Bar graphs are presented as mean ± SEM. (**A**,**B**): statistical differences were measured using One-way ANOVA and Tukey’s multiple comparison test. (**C**,**D**): statistical differences were measured using unpaired Student’s *t*-test. n.s. indicates no significant difference, * *p* < 0.05, ** *p* < 0.01, *** *p* < 0.001, **** *p*< 0.0001. Each experiment was conducted in triplicate.

In order to identify whether the sleep phenotypes of the *14-3-3ε* mutant are related to these genes, we first measured sleep phenotypes of the *14-3-3ε* mutant and receptor mutants of the neurotransmitters *Dop1R1* and *Oamb*, respectively. The results showed that sleep decreased in the *14-3-3ε* mutant but increased in the *Dop1R1* and *Oamb* receptor mutants compared to their controls (Figure 7A,B). Furthermore, we examined the genetic interactions between *14-3-3ε* and the *Dop1R1* or *Oamb* receptor by using the flies of simultaneously mutating *14-3-3ε* and *Dop1R1* (*Dop1R1^KO^* /*14-3-3ε^j2B10^*) or *14-3-3ε* and *Oamb* (*Oamb^M111578^/14-3-3ε^j2B10^*). The results showed that decreases in sleep phenotype caused by *14-3-3ε^j2B10^*/+ could be partially recovered in these double-mutant flies (Figure 7A,B).

Does *14-3-3ε* regulate PDF by these neurotransmitters? To answer this question, we analyzed the relationship by detecting PDF levels in the double-mutant flies of *14-3-3ε* and *Dop1R1* (*Dop1R1^KO^*/*14-3-3ε^EP3578^*) or *14-3-3ε* and *Oamb* (*Oamb^M111578^/14-3-3ε^EP3578^*). The results showed that decreases in PDF level caused by *14-3-3ε* mutant flies could be partially recovered in these double-mutant flies (Figure 7C,D), which is a similar finding to that regarding the sleep behaviors presented in Figure 7A,B. These results indicate that the sleep phenotypes of the *14-3-3ε* mutant are related to these neurotransmitters.

**Figure 8:**
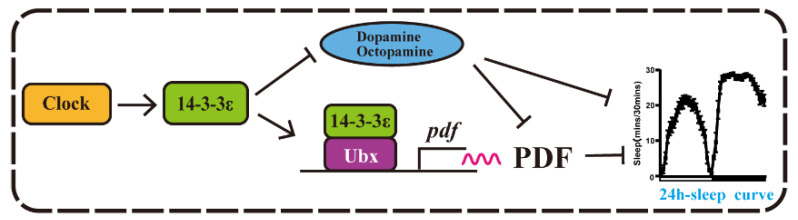
The 14-3-3ε mediated sleep regulation in Drosophila: A model shows that 14-3-3ε regulates sleep through Ubx-PDF pathways and neurotransmitters.

From all of the above data, we propose a model for *14-3-3ε* sleep regulation. The *14-3-3ε* protein regulates sleep through two pathways: one is achieved by regulating PDF pathway through interacting with Ubx, which results in a negative regulation of sleep; on the other hand, *14-3-3ε* regulates the synthesis enzymes of the neurotransmitters, which results in positive regulation of sleep. As a result, *14-3-3ε* integrates these factors to maintain a sleep balance (Figure 8).

## 4. Discussion

The pigment dispersing factor (PDF), a neuropeptide secreted from the LNv neurons of the brain, is a wake-promoting factor. When flies are stimulated by light, LNvs respond to light and promote arousal by releasing PDF [28,29]. Loss of PDF leads to an increase in the amount of sleep in Drosophila [30]. Functionally, it is analogous to vertebrate orexin/hypocretin [30,31,32]. In mammals, the neuropeptide vasoactive intestinal peptide (VIP) functions to synchronize the oscillations of clock neurons and transfer circadian signals to downstream neurons [33,34].

Ultrabithorax (Ubx) encodes a homeodomain transcription factor involved in cell fate decisions, cell proliferation, and organ identity, and it belongs to the Hox gene family. Hox genes, including Sex-combs reduced (Scr), Antennapedia (Antp), Ultrabithorax (Ubx) and abdominal-A (abd-A), play a conserved role in establishing the thoracic and abdominal segments during insect embryogenesis [35]. Singh et al. reported that Ubx regulates the Fat/Hippo and IIS/dAkt pathways in specifying haltere development, including organ decision and size, sensory bristle repression, trichome morphology, and arrangement. The Ubx-mediated Fat/Hippo pathway is key for the transformation of wing identity to haltere [36]. Ubx functions as a tumor inhibitor in its respective endogenous domains [37]. When interacting with Pho, Ubx can stabilize lineage choice through suppressing the multipotency encoded in the genome [38]. Regulated by polycomb complex, Ubx is a repressor of alternative cell fates within the mesoderm, and it also maintains normal muscle differentiation by repressing Twi [39]. In this study, Ubx takes part in regulating fly sleep by cooperating with *14-3-3ε*. 

In this study, we identified the role of *14-3-3ε* in sleep regulation. *14-3-3ε,* controlled by *Clock*, regulates both PDF and metabolic factors that are important for neurotransmitter biogenesis. Previous studies showed that multiple types of neurotransmitters had been identified, including acetylcholine (Ach), noradrenaline (NA), histamine, 5-hydroxytryptophan (5-HT), dopamine (DA), glutamate (Glu), and γ-aminobutyric acid (GABA) [22]. The production of many neurotransmitters is closely related to amino acid production. Glutamate (glutamic acid) is a natural amino acid, while GABA (γ-amino butyric acid) is derived from glutamate. Serotonin (also called 5-HT), dopamine, noradrenaline, and histamine are derived from aromatic amino acids like tyrosine and belong to the monoamine neurotransmitters. Our data from the RNA-seq indicate that multiple factors in the tyrosine and glutamate metabolic process are affected in the *14-3-3ε* mutant, among which dopamine and octopamine have been proved to be sleep regulators by previous studies in *Drosophila*. Thus, *14-3-3ε* was found to be a novel regulator of neurotransmitters in this study, in which the mutants from the *Dop1R1* and *Oamb* can restore the *14-3-3ε* phenotype to different degrees. In this study, a new sleep regulation pathway, i.e., *14-3-3ε,* that regulates sleep through the dopamine and octopamine signal pathway, was identified.

*14-3-3* proteins are found to be important in both cancer- and age-related neurodegenerative disease [40], which can directly interact with *yki* [17], an important cancer factor in the Hippo pathway [19,40]. Previous studies indicated that *14-3-3ε* is involved in gastric cancer and colorectal cancer [41,42,43,44,45,46], and disrupted sleep is a risk factor that contributes to cancer [47]. Many current papers showed a link between molecules upregulated in cancer patients and selected sleep disturbances. The obstructive sleep apnea (OSA) patients have less sleep and worse sleep quality, in which the serum hypoxia-inducible factor 1α (HIF-1α) protein level as a key factor of cellular oxygen metabolism is significantly higher [48,49,50,51,52]. Hypoxia is regarded as a feature of rapidly proliferating tissues, such as cancer [53]. HIF-1αof dysregulation/overexpression have been connected to both obstructive sleep apnea and cancer biology, specifically in areas of vascularization and angiogenesis, energy metabolism, cell survival, tumor invasion, and so on [48,49,50,51]. This link is interesting because disruption of HIF-1α expression may lead to a developing circadian clock disruption, as its increased protein level is associated with overexpression of circadian clock proteins [54].

As is well known, the clock genes regulate the circadian rhythm or/and sleep [2]. In this current study, we found that *14-3-3ε,* controlled by Clock, regulates sleep through pathways of both the 14-3-3ε/Ubx/PDF and neurotransmitters. In addition, there are previous reports that *14-3-3ε* is also related to cancers [40,41,42,43,44,45,46], indicating that *14-3-3ε* is a multifunctional gene in Drosophila regulating different physiological activities. Currently, the direct relationship between cancer and sleep is still unclear, which need to be carefully designed for systematical investigation in future. 

## Data Availability

The RNA-seq data from this publication have been deposited in the NCBI bioproject database (https://www.ncbi.nlm.nih.gov/bioproject/) (accessed on 1 May 2021) and assigned the identifier PRJNA680635.

## Supplementary Materials

The following are available online at https://www.mdpi.com/article/10.3390/ijms22189748/s1.
