# Supplementary material for: Integrative Role of 14-3-3ε in Sleep Regulation
Source: Int J Mol Sci. 2021 Sep 9;22(18):9748. doi: 10.3390/ijms22189748 (PMC8467329; doi:10.3390/ijms22189748)
Supplement: Supplementary file 1 [file ijms-22-09748-s001.zip › Figure S1.pdf]

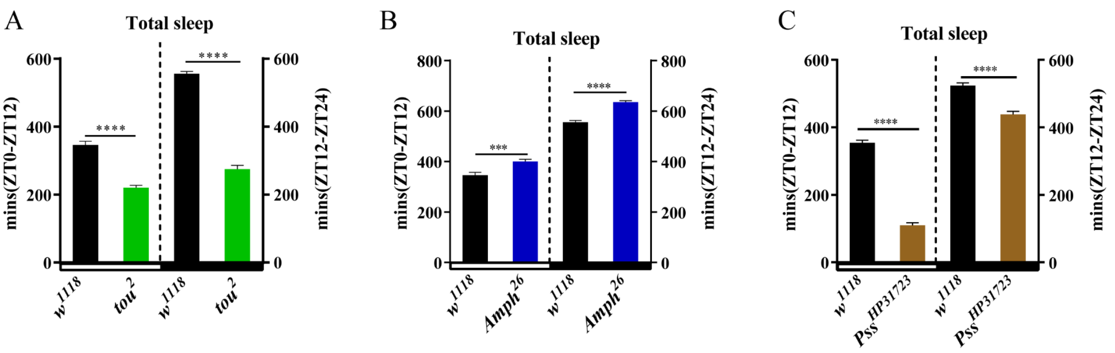

Figure S1. Sleep patterns of candidate gene mutants.

A, Sleep pattern of *tou* mutant. B, Sleep pattern of *Amph* mutant. C, Sleep pattern of *Pss* mutant. Statistical differences were measured using unpaired Student's t-test; ***P <0.001, ****P<0.0001. Each experiment was conducted in triplicate.
