# Supplementary material for: Integrative Role of 14-3-3ε in Sleep Regulation
Source: Int J Mol Sci. 2021 Sep 9;22(18):9748. doi: 10.3390/ijms22189748 (PMC8467329; doi:10.3390/ijms22189748)
Supplement: Supplementary file 1 [file ijms-22-09748-s001.zip › Table S2 Transcriptome different gene list at ZT2 and ZT14 20210722.pdf]

#ID	gene_name	w^{1118}_ZT2_Count	*14-3-3ε*^{EP3578}_ZT2_Count	w^{1118}_ZT2_FPKM	*14-3-3ε*^{EP3578}_ZT2_FPKM	FDR	log2FC	regulated
FBgn0029881	pigs	3571	2480	33.4021	21.9852	0.0020	-0.5271	down
FBgn0043470	lambdaTry	0	18	0.0000	1.6104	0.0062	4.1478	up
FBgn0011770	Gip	1165	1800	98.0954	152.6030	0.0001	0.6259	up
FBgn0039325	CG10560	479	2079	23.5643	97.9310	0.0000	2.1140	up
FBgn0036571	Strump	829	471	13.7628	7.9442	0.0000	-0.8156	down
FBgn0039073	CG4408	380	770	16.3082	34.0199	0.0000	1.0155	up
FBgn0040465	Dlip3	362	219	17.5822	10.8525	0.0022	-0.7236	down
FBgn0037375	kat-60L1	1262	861	34.8507	25.3095	0.0030	-0.5524	down
FBgn0036105	Blos4	120	38	13.1248	4.9212	0.0000	-1.6329	down
FBgn0038739	CG4686	259	466	24.7334	45.7935	0.0000	0.8434	up
FBgn0030296	CG15196	84	12	3.6581	0.5398	0.0000	-2.7031	down
FBgn0037713	CG16790	797	1443	73.1106	109.1563	0.0000	0.8542	up
FBgn0250871	pot	922	1354	13.4312	20.0011	0.0025	0.5525	up
FBgn0087007	bbg	1092	718	13.2763	9.4390	0.0006	-0.6055	down
FBgn0032726	CG10621	497	737	34.9561	53.8525	0.0083	0.5661	up
FBgn0033820	CG4716	13778	9527	1669.2534	1148.5406	0.0017	-0.5336	down
FBgn0030674	HUWE1	4492	3169	16.6340	12.2500	0.0049	-0.5045	down
FBgn0259145	CG42260	1463	586	11.0041	4.4129	0.0000	-1.3197	down
FBgn0038840	Grik	584	352	11.5918	7.3038	0.0001	-0.7300	down
FBgn0011722	Tig	2113	1421	17.5206	12.5103	0.0005	-0.5734	down
FBgn0039543	CROT	1316	433	39.6921	13.5055	0.0000	-1.6027	down
FBgn0016013	Faa	550	1166	28.4124	61.0294	0.0000	1.0812	up
FBgn0085261	CG34232	6	45	0.9082	5.8680	0.0000	2.7018	up
FBgn0027584	CG4757	715	1339	25.8686	48.1855	0.0000	0.9028	up
FBgn0069969	CG40498	2090	3497	63.9237	84.7319	0.0000	0.7410	up
FBgn0029879	APC7	66	15	2.0122	0.4650	0.0000	-2.0622	down
FBgn0032264	Lip4	993	354	44.7439	14.6226	0.0000	-1.4866	down
FBgn0037687	CG8132	283	457	22.1731	36.6154	0.0016	0.6880	up
FBgn0031432	Cyp309a1	1339	576	49.4685	22.4648	0.0000	-1.2168	down
FBgn0034140	Lst	861	1947	53.8918	126.0016	0.0000	1.1748	up
FBgn0038302	CG4210	100	40	16.1480	6.4326	0.0011	-1.3004	down
FBgn0013279	Hsp70Bc	108	15	2.7173	0.4098	0.0000	-2.7637	down
FBgn0034480	CG16898	733	352	41.8774	19.3457	0.0000	-1.0573	down
FBgn0034639	CG15673	201	373	14.5938	27.9041	0.0000	0.8871	up
FBgn0030313	Reepl1	101	0	9.7972	0.0000	0.0000	-6.5689	down
FBgn0013772	Cyp6a8	539	1711	19.6073	61.2606	0.0000	1.6632	up
FBgn0037537	CG2767	2602	3774	130.5800	193.6440	0.0014	0.5349	up
FBgn0051183	CG31183	1097	599	12.8976	7.3403	0.0000	-0.8731	down
FBgn0019928	Ser8	0	21	0.0000	2.2876	0.0008	4.3585	up
FBgn0034745	CG4329	13	52	0.2169	0.8491	0.0013	1.9136	up
FBgn0033982	Cyp317a1	168	329	4.6326	7.1020	0.0000	0.9638	up
FBgn0035106	rno	1808	1110	8.4906	5.1096	0.0000	-0.7046	down
FBgn0036022	CG8329	19179	47716	2013.0600	5161.1300	0.0000	1.3136	up
FBgn0063497	GstE3	901	1882	116.8840	252.7220	0.0000	1.0604	up
FBgn0259209	Mlp60A	1680	2561	275.9287	506.1219	0.0001	0.6066	up
FBgn0029924	CG4586	64	182	1.9597	6.1148	0.0000	1.4909	up
FBgn0052037	CG32037	76	23	3.1529	0.9272	0.0002	-1.6800	down
FBgn0015570	alpha-Est2	986	561	33.0890	18.9776	0.0000	-0.8137	down
FBgn0039686	CG15506	38	183	1.7571	8.1761	0.0000	2.2346	up
FBgn0037081	barc	1237	1835	34.1792	50.4108	0.0008	0.5672	up
FBgn0029837	Tsp5D	959	636	37.6702	24.4933	0.0014	-0.5930	down
FBgn0031080	CG12655	349	43	61.8349	7.8641	0.0000	-2.9909	down
FBgn0037630	Ir85a	59	17	1.8936	0.5659	0.0016	-1.7340	down
FBgn0028999	nerfin-1	18	0	0.2871	0.0000	0.0061	-4.1491	down
FBgn0032066	LManIII	3	48	0.0821	0.9766	0.0000	3.5888	up
FBgn0050263	stum	147	271	2.1418	4.4290	0.0003	0.8763	up
FBgn0035665	Jon65Aiii	0	21	0.0000	2.1813	0.0008	4.3585	up
FBgn0267398	Yeti	942	1531	87.2676	128.8519	0.0000	0.6987	up
FBgn0086558	Ubi-p5E	9270	3548	270.2286	102.7222	0.0000	-1.3866	down
FBgn0023178	Pdf	1736	1192	239.7200	170.6630	0.0022	-0.5433	down
FBgn0033397	Cyp4p3	199	93	8.1636	3.7173	0.0000	-1.0900	down
FBgn0036121	CG6310	21	91	3.2439	14.0486	0.0000	2.0591	up
FBgn0014859	Hr38	278	654	2.4137	5.5864	0.0000	1.2297	up
FBgn0036381	CG8745	2473	852	69.7948	23.7752	0.0000	-1.5375	down
FBgn0051955	CG31955	68	5	10.5314	0.4866	0.0000	-3.5083	down
FBgn0038339	CG6118	0	63	0.0162	1.0796	0.0000	5.8958	up
FBgn0037171	CG14459	35	1	1.9756	0.0540	0.0000	-4.1204	down
FBgn0034356	Pepck2	91	1187	2.3294	33.0486	0.0000	3.6883	up
FBgn0036752	Adgf-A	819	518	21.3694	14.2762	0.0002	-0.6612	down
FBgn0036254	CG5645	165	88	3.2072	1.6674	0.0083	-0.9001	down
FBgn0032647	CG15143	25	69	0.3993	1.0377	0.0074	1.4249	up
FBgn0032973	CG6675	87	211	4.5836	11.0993	0.0000	1.2664	up
FBgn0038142	CheA87a	447	233	69.3445	35.6689	0.0000	-0.9381	down
FBgn0040323	GNBP1	560	259	20.7615	9.8100	0.0000	-1.1106	down
FBgn0050088	CG30088	109	12	8.8748	1.0107	0.0000	-3.0748	down
FBgn0036619	Cpr72Ec	3455	13779	130.0090	538.1560	0.0000	1.9940	up
FBgn0014342	mia	158	58	4.9366	1.7987	0.0000	-1.4304	down
FBgn0033942	Cpr51A	106	2	16.6952	0.3233	0.0000	-5.1227	down
FBgn0030607	dob	601	1011	21.5797	37.8347	0.0000	0.7480	up
FBgn0000044	Act57B	9841	21555	454.5960	1043.7500	0.0000	1.1297	up
FBgn0032817	CG10631	2552	1733	12.2745	8.5344	0.0007	-0.5594	down
FBgn0035890	CG13667	139	62	5.2405	2.3448	0.0004	-1.1524	down
FBgn0042175	CG18858	266	461	9.9854	17.5401	0.0001	0.7895	up
FBgn0032900	CG14401	560	325	39.9338	23.1150	0.0000	-0.7843	down
FBgn0035550	CG11349	64	0	2.3946	0.0000	0.0000	-5.9195	down
FBgn0032281	CG17107	50	266	21.9546	117.7070	0.0000	2.3852	up
FBgn0033158	CG12164	2	39	0.1477	1.8281	0.0000	3.7027	up
FBgn0039685	Obp99b	11468	4824	2495.6428	1069.7111	0.0000	-1.2505	down
FBgn0262870	axo	2012	1303	16.0562	10.3561	0.0000	-0.6277	down
FBgn0000046	Act87E	1555	2559	77.0535	127.7096	0.0000	0.7169	up
FBgn0032144	CG17633	0	37	0.0528	2.0902	0.0000	5.1449	up

FBgn0262150	CG42876	128	3	6.3643	0.1179	0.0000	-4.9862	down
FBgn0259236	comm3	378	233	12.4940	8.4875	0.0035	-0.6968	down
FBgn0039722	Capa	1685	978	234.4730	138.0200	0.0000	-0.7855	down
FBgn0034436	CG11961	711	472	16.0654	10.8973	0.0042	-0.5913	down
FBgn0039161	CG13606	434	241	37.3402	23.0170	0.0000	-0.8471	down
FBgn0038056	CG5961	572	294	27.0117	14.1886	0.0000	-0.9590	down
FBgn0042127	CG18789	45	7	2.5250	0.3998	0.0001	-2.5136	down
FBgn0038292	CG3987	14	85	0.7299	4.2519	0.0000	2.5121	up
FBgn0003863	alphaTry	0	32	0.0000	3.9149	0.0000	4.9418	up
FBgn0035941	CG13313	44	4	2.5389	0.2439	0.0000	-3.1519	down
FBgn0034069	CG8401	653	397	53.5466	34.6411	0.0001	-0.7178	down
FBgn0013813	Dhc98D	41	391	0.1787	1.6760	0.0000	3.2188	up
FBgn0032181	CG13133	25	69	1.6867	4.8799	0.0074	1.4249	up
FBgn0034329	BomS1	689	346	371.8690	189.0760	0.0000	-0.9928	down
FBgn0033186	CG1602	60	149	2.0809	5.1723	0.0000	1.2957	up
FBgn0031528	CG15412	200	326	7.9338	13.3549	0.0071	0.7005	up
FBgn0052029	Cpr66D	579	102	11.2172	3.2664	0.0000	-2.4939	down
FBgn0042178	Apl	69	237	1.2079	4.1856	0.0000	1.7631	up
FBgn0038682	CG5835	265	540	14.7145	30.6424	0.0000	1.0227	up
FBgn0033205	CG2064	313	182	20.3255	11.8867	0.0013	-0.7800	down
FBgn0035382	Or63a	139	67	5.8196	2.8522	0.0027	-1.0423	down
FBgn0003082	phr	51	816	2.0232	31.2445	0.0000	3.9705	up
FBgn0053548	SmydA-8	3133	4697	121.0377	199.5461	0.0001	0.5827	up
FBgn0029826	CG6041	365	559	8.7375	8.6414	0.0056	0.6121	up
FBgn0036725	CG18265	384	225	2.5527	1.3988	0.0004	-0.7697	down
FBgn0004629	Cys	3980	7266	1259.7400	2343.2700	0.0000	0.8669	up
FBgn0039752	CG15530	2	26	0.0889	0.8346	0.0025	3.1369	up
FBgn0053329	Sp212	476	735	20.3062	32.3395	0.0013	0.6243	up
FBgn0036145	CG7607	662	356	87.8140	46.6865	0.0000	-0.8943	down
FBgn0038465	Irc	2947	1833	83.1667	51.6056	0.0000	-0.6861	down
FBgn0034629	Acox57D-d	262	86	7.6934	2.5724	0.0000	-1.5965	down
FBgn0033518	Prx2540-2	611	329	55.9104	31.6071	0.0000	-0.8923	down
FBgn0036290	CG10638	609	1241	38.4565	78.5246	0.0000	1.0244	up
FBgn0259682	Jabba	4895	1875	212.6663	79.0560	0.0000	-1.3852	down
FBgn0002571	Mal-A3	0	45	0.0353	1.7118	0.0000	5.4201	up
FBgn0035766	eco	678	1745	11.5899	30.2146	0.0000	1.3611	up
FBgn0033065	Cyp6w1	4210	11105	156.1575	407.8802	0.0000	1.3977	up
FBgn0010403	Obp83b	42	120	10.5004	29.8506	0.0000	1.4896	up
FBgn0015035	Cyp4e3	805	151	31.6097	5.8607	0.0000	-2.4075	down
FBgn0053138	AGBE	2379	3438	68.2157	104.0690	0.0019	0.5297	up
FBgn0037607	CG8036	9407	15378	281.2952	487.9850	0.0000	0.7077	up
FBgn0000500	Dsk	1675	940	355.9130	201.0830	0.0000	-0.8340	down
FBgn0023458	Rbcn-3A	6815	4757	38.1702	28.0174	0.0025	-0.5199	down
FBgn0040349	CG3699	1002	3155	101.2510	330.7170	0.0000	1.6524	up
FBgn0034564	CG9344	44	103	15.1596	34.2083	0.0030	1.2059	up
FBgn0031542	CG15414	202	109	10.2177	5.5671	0.0026	-0.8849	down
FBgn0028983	Spn55B	433	165	23.2713	9.3056	0.0000	-1.3874	down
FBgn0037030	CG3288	31	1	2.7071	0.1171	0.0000	-3.9508	down
FBgn0038160	CG9759	790	275	53.1840	17.5701	0.0000	-1.5201	down
FBgn0033387	CG8008	513	853	13.2791	21.2922	0.0000	0.7310	up
FBgn0039925	Kif3C	483	858	5.9615	8.6065	0.0000	0.8262	up
FBgn0023507	D2hgdh	4283	9349	133.6825	309.4173	0.0000	1.1247	up
FBgn0004795	retn	860	1245	12.5901	21.5887	0.0064	0.5318	up
FBgn0034606	ASPP	1380	2011	17.8510	26.8826	0.0019	0.5416	up
FBgn0032266	CG18302	990	658	32.2935	20.7179	0.0015	-0.5899	down
FBgn0013348	TpnC41C	156	418	16.6014	46.6829	0.0000	1.4144	up
FBgn0038981	CG5346	787	1203	24.7115	39.3614	0.0004	0.6102	up
FBgn0024289	Sodh-1	4090	6767	262.4660	464.5393	0.0000	0.7249	up
FBgn0033067	CG11211	1437	3332	287.7640	658.9130	0.0000	1.2114	up
FBgn0054054	CG34054	159	50	34.0196	10.6587	0.0000	-1.6494	down
FBgn0040211	hgo	1068	1645	51.7054	81.8205	0.0001	0.6213	up
FBgn0034660	Loxl2	272	467	10.9446	19.5408	0.0001	0.7761	up
FBgn0010038	GstD2	35	110	4.5847	14.4564	0.0000	1.6211	up
FBgn0043783	CG32444	6859	3364	367.7370	186.7000	0.0000	-1.0289	down
FBgn0263780	CG17684	722	441	11.6896	6.2590	0.0001	-0.7112	down
FBgn0264489	CG43897	8295	13006	199.6826	303.4444	0.0000	0.6475	up
FBgn0030300	Sk1	283	450	7.3879	11.9499	0.0033	0.6658	up
FBgn0037930	CG14715	194	76	29.9678	11.8881	0.0000	-1.3410	down
FBgn0042177	Arts	703	387	11.7133	6.4994	0.0000	-0.8607	down
FBgn0034162	CG6426	4090	2867	656.4420	471.0250	0.0034	-0.5137	down
FBgn0035207	Herc4	3658	5961	63.1895	106.0335	0.0000	0.7030	up
FBgn0033574	Spn47C	456	1002	22.0263	44.9291	0.0000	1.1326	up
FBgn0261041	stj	7172	4018	75.2686	42.4242	0.0000	-0.8371	down
FBgn0040732	CG16926	3396	902	286.6340	72.8977	0.0000	-1.9127	down
FBgn0027655	htt	3157	1528	29.1928	19.8468	0.0000	-1.0477	down
FBgn0053105	p24-2	6	1161	0.1396	61.1201	0.0000	7.3584	up
FBgn0031367	c-cup	29	0	2.8343	0.0000	0.0000	-4.8060	down
FBgn0086698	frtz	322	502	5.6364	9.1527	0.0043	0.6376	up
FBgn0027548	nito	642	935	10.0404	15.2462	0.0094	0.5403	up
FBgn0050083	CG30083	27	87	2.6179	7.6579	0.0000	1.6481	up
FBgn0013812	Dhc93AB	274	131	1.1125	0.5496	0.0000	-1.0598	down
FBgn0036877	CG9452	50	143	2.0309	5.9065	0.0000	1.4948	up
FBgn0052726	CG32726	665	418	784.1781	481.4256	0.0004	-0.6698	down
FBgn0085256	CG34227	933	1527	268.6460	452.2960	0.0000	0.7088	up
FBgn0033999	CG8093	0	25	0.0000	1.5630	0.0001	4.5988	up
FBgn0000053	Gart	1571	3402	26.8525	54.0616	0.0000	1.1128	up
FBgn0027620	Acf	678	163	7.7388	1.8397	0.0000	-2.0505	down
FBgn0067779	dbr	935	641	19.5040	8.9281	0.0080	-0.5452	down
FBgn0034471	Obp56e	512	1385	123.2282	332.3696	0.0000	1.4324	up
FBgn0051036	CG31036	282	582	5.1029	10.8986	0.0000	1.0412	up
FBgn0001128	Gpdh1	17469	11513	693.3923	458.3391	0.0001	-0.6028	down
FBgn0034808	CG9896	807	1205	24.4828	36.1885	0.0014	0.5764	up

FBgn0000047	Act88F	38	198	1.7534	9.6438	0.0000	2.3477	up
FBgn0040364	CG11378	1539	668	85.3192	39.0547	0.0000	-1.2041	down
FBgn0085209	CG34180	65	144	37.4562	80.7981	0.0004	1.1333	up
FBgn0004797	mdy	1197	736	40.6398	25.7333	0.0000	-0.7022	down
FBgn0035725	Mis12	74	16	7.8113	1.7110	0.0000	-2.1377	down
FBgn0030600	hiw	5945	3629	21.9381	14.1450	0.0000	-0.7133	down
FBgn0036837	CG18135	13097	8025	289.7549	174.9522	0.0000	-0.7079	down
FBgn0033354	FANCI	176	25	2.8490	0.3866	0.0000	-2.7649	down
FBgn0029821	CG4020	1	21	0.0756	0.8535	0.0084	3.4099	up
FBgn0038702	CG3739	0	39	0.0000	1.8069	0.0000	5.2188	up
FBgn0031375	erm	291	451	6.2188	10.2520	0.0088	0.6289	up
FBgn0015039	Cyp9b2	3027	4651	122.0580	190.9370	0.0000	0.6181	up
FBgn0036757	Ir75a	272	7	9.4362	0.2470	0.0000	-5.0808	down
FBgn0031805	Nepl4	247	113	7.3740	3.5597	0.0000	-1.1221	down
FBgn0260793	2mit	2433	1664	15.2284	9.7697	0.0011	-0.5491	down
FBgn0040817	CG14132	311	517	28.4673	46.9600	0.0002	0.7299	up
FBgn0002543	robo2	356	198	4.6614	3.8703	0.0001	-0.8442	down
FBgn0031169	CG1494	172	14	2.0865	0.1765	0.0000	-3.5224	down
FBgn0033204	CG2065	262	671	16.8974	44.1138	0.0000	1.3518	up
FBgn0025583	BomS2	1911	380	1072.7200	211.1330	0.0000	-2.3283	down
FBgn0038820	CG4000	3087	1300	253.0850	118.0500	0.0000	-1.2483	down
FBgn0032507	CG9377	586	1361	41.5984	98.7628	0.0000	1.2128	up
FBgn0035258	CG13931	10	68	1.4580	10.3575	0.0000	2.6395	up
FBgn0044050	Ilp3	88	13	9.9453	1.5206	0.0000	-2.6631	down
FBgn0086348	se	2219	1531	248.4800	176.0520	0.0021	-0.5365	down
FBgn0046114	Gclm	1803	1031	108.5884	66.6925	0.0000	-0.8071	down
FBgn0034599	hng1	115	213	7.8870	15.1687	0.0019	0.8817	up
FBgn0036289	CG10657	96	187	4.2537	8.3993	0.0012	0.9528	up
FBgn0035205	Ctr9	1659	3950	25.7396	63.1953	0.0000	1.2497	up
FBgn0025814	Mgstl	1434	989	218.7045	155.4129	0.0040	-0.5369	down
FBgn0030187	Ipod	98	11	5.3911	0.6275	0.0000	-3.0377	down
FBgn0033636	tou	4493	3099	31.5812	27.8281	0.0012	-0.5371	down
FBgn0052698	CARPB	1134	548	26.1728	5.6728	0.0000	-1.0491	down
FBgn0000163	baz	2981	1731	16.6409	11.4804	0.0000	-0.7852	down
FBgn0000527	e	802	521	16.6858	11.2427	0.0010	-0.6226	down
FBgn0033926	Arc1	10925	7491	284.3240	194.2460	0.0008	-0.5457	down
FBgn0032189	Ripalpha	142	271	8.0634	14.6028	0.0001	0.9259	up
FBgn0036024	CG18180	11	57	1.1045	5.7146	0.0000	2.2646	up
FBgn0036862	Gbs-76A	3382	1912	53.5519	27.6448	0.0000	-0.8238	down
FBgn0040606	CG6503	7491	4875	6672.3200	4166.9403	0.0000	-0.6210	down
FBgn0037845	CG14694	232	108	13.2133	6.4561	0.0000	-1.0968	down
FBgn0051778	CG31778	925	589	65.5724	44.4857	0.0002	-0.6516	down
FBgn0034931	CG2812	118	213	6.8662	13.0262	0.0040	0.8449	up
FBgn0034407	DptB	9	53	3.3854	18.4973	0.0000	2.4228	up
FBgn0039068	CG13827	35	112	3.2737	9.6703	0.0000	1.6469	up
FBgn0012042	AttA	111	345	10.5965	33.4233	0.0000	1.6253	up
FBgn0037292	plh	88	251	11.9645	40.0002	0.0000	1.4994	up
FBgn0036589	CG13067	149	67	27.0082	12.8077	0.0003	-1.1418	down
FBgn0035670	CG10472	24	184	2.3717	18.4327	0.0000	2.8824	up
FBgn0085485	CG34456	11	199	2.2663	38.2055	0.0000	4.0491	up
FBgn0004102	oc	703	1049	9.3839	13.2156	0.0021	0.5753	up
FBgn0032699	CG10383	448	768	10.1345	17.4833	0.0000	0.7748	up
FBgn0028920	CG8997	8	70	0.5071	6.6169	0.0000	2.9680	up
FBgn0040256	Ugt35C1	629	1572	28.1477	69.3495	0.0000	1.3187	up
FBgn0016131	Cdk4	173	310	7.4607	11.9585	0.0003	0.8362	up
FBgn0003996	w	925	1344	38.9273	50.8338	0.0046	0.5371	up
FBgn0029755	Sas10	152	270	6.0070	11.1159	0.0012	0.8231	up
FBgn0030098	CG12057	0	61	0.0000	6.6146	0.0000	5.8501	up
FBgn0035918	Cdc6	169	10	4.3059	0.2730	0.0000	-3.9420	down
FBgn0033229	CG12822	1159	733	58.2756	34.6050	0.0001	-0.6616	down
FBgn0038291	CG3984	118	1068	6.0139	56.3537	0.0000	3.1651	up
FBgn0031021	ND-18	1527	2195	161.6550	239.1960	0.0038	0.5219	up
FBgn0036449	bmm	1078	1581	30.4126	45.2441	0.0020	0.5507	up
FBgn0042712	HBS1	304	656	8.6170	19.5077	0.0000	1.1056	up
FBgn0036992	Hpd	7085	13401	408.5577	805.1390	0.0000	0.9181	up
FBgn0051313	CG31313	4715	3160	1062.8244	662.0841	0.0002	-0.5785	down
FBgn0044810	TotX	1074	333	238.6320	71.9409	0.0000	-1.6875	down
FBgn0037298	Sccpdh1	6679	3837	279.4793	168.2048	0.0000	-0.8008	down
FBgn0030159	CG9689	1130	675	137.8292	67.4909	0.0000	-0.7438	down
FBgn0039678	Obp99a	46	6	10.5784	1.4264	0.0000	-2.7353	down
FBgn0052170	CG32170	269	421	8.6574	14.1465	0.0083	0.6428	up
FBgn0031860	CG11236	284	532	18.6948	35.4569	0.0000	0.9017	up
FBgn0010385	Def	31	271	15.1127	135.1410	0.0000	3.0831	up
FBgn0034741	CG4269	6	42	0.7220	6.9736	0.0001	2.6047	up
FBgn0036278	CrzR	405	632	9.2189	13.9630	0.0015	0.6393	up
FBgn0032414	CG17211	1069	719	13.9050	9.2603	0.0022	-0.5728	down
FBgn0030349	CG10353	3178	2202	42.7812	30.8910	0.0019	-0.5304	down
FBgn0025454	Cyp6g1	6774	12139	237.8640	442.1795	0.0000	0.8401	up
FBgn0051469	CG31469	34	144	6.9699	28.6231	0.0000	2.0469	up
FBgn0003048	pcx	1212	781	6.2034	4.1318	0.0001	-0.6346	down
FBgn0036436	CG4914	55	3	2.3197	0.1302	0.0000	-3.7834	down
FBgn0051089	CG31089	20	0	1.0707	0.0000	0.0016	-4.2929	down
FBgn0030773	CG9676	11335	16501	874.9630	1341.4300	0.0013	0.5404	up
FBgn0031313	CG5080	2001	3363	60.5371	109.7544	0.0000	0.7474	up
FBgn0023550	FarO	301	155	8.2955	5.1751	0.0000	-0.9540	down
FBgn0035206	sturkopf	494	872	24.5899	44.4856	0.0000	0.8171	up
FBgn0033729	Cpr49Af	9	43	2.1323	9.9346	0.0019	2.1279	up
FBgn0036806	Cyp12c1	712	372	24.9699	13.4524	0.0000	-0.9359	down
FBgn0039896	yellow-h	450	255	21.7200	11.3494	0.0000	-0.8181	down
FBgn0035282	CNMa	106	20	4.2419	0.8806	0.0000	-2.3463	down
FBgn0032069	LManVI	2	308	0.0617	6.7758	0.0000	6.6498	up
FBgn0036585	CG13071	142	55	45.4326	17.9434	0.0000	-1.3527	down

FBgn0036875	CG9449	440	878	19.5052	33.1115	0.0000	0.9936	up
FBgn0037975	CG3397	445	906	26.8680	57.4793	0.0000	1.0226	up
FBgn0003499	sr	276	501	3.7637	5.9224	0.0000	0.8563	up
FBgn0041579	AttC	84	204	6.9325	17.3719	0.0000	1.2680	up
FBgn0051773	CG31773	29	4	0.6472	0.0883	0.0081	-2.5681	down
FBgn0036597	CG4962	593	44	68.6784	5.3208	0.0000	-3.7216	down
FBgn0039312	CG10514	134	238	7.7277	13.8210	0.0030	0.8224	up
FBgn0031327	CG5397	919	1400	27.5793	41.8372	0.0003	0.6054	up
FBgn0035262	CG18171	110	49	2.5880	1.1717	0.0036	-1.1507	down
FBgn0026314	Ugt35B1	589	3952	24.5399	163.4580	0.0000	2.7427	up
FBgn0039684	Obp99d	2	51	0.8979	15.3532	0.0000	4.0805	up
FBgn0026593	CG5707	1469	816	69.5992	40.4077	0.0000	-0.8487	down
FBgn0028984	Spn88Ea	1353	1945	58.4018	86.8530	0.0044	0.5219	up
FBgn0030027	CG1632	407	671	6.1390	10.6881	0.0001	0.7184	up
FBgn0027611	LManII	8545	5728	159.2929	107.5585	0.0002	-0.5783	down
FBgn0013949	Elal	1084	1567	71.8223	111.9076	0.0045	0.5299	up
FBgn0038290	CG6912	74	2656	3.4504	87.3748	0.0000	5.1440	up
FBgn0034491	Hsl	1203	520	22.8576	10.3866	0.0000	-1.2097	down
FBgn0031746	CG9029	0	28	0.0000	5.9067	0.0000	4.7559	up
FBgn0011279	Obp69a	11	73	3.2127	18.8603	0.0000	2.6156	up
FBgn0028919	CG16865	101	196	7.6696	15.4503	0.0009	0.9478	up
FBgn0051324	CG31324	373	631	12.3752	19.0856	0.0000	0.7554	up
FBgn0261402	Ir75b	180	3	6.7464	0.1146	0.0000	-5.4746	down
FBgn0266064	GlyS	2394	3466	61.4620	92.3479	0.0016	0.5323	up
FBgn0263772	CG43689	584	373	6.1657	4.0326	0.0016	-0.6466	down
FBgn0031454	HemK2	336	549	19.2447	33.2017	0.0003	0.7052	up
FBgn0038194	Cyp6d5	4980	8015	207.3020	331.0360	0.0000	0.6851	up
FBgn0013307	Odc1	1098	365	62.1176	21.3938	0.0000	-1.5874	down
FBgn0013278	Hsp70Bb	74	22	1.9395	0.6197	0.0002	-1.7033	down
FBgn0052104	CG32104	291	749	4.4110	10.2277	0.0000	1.3594	up
FBgn0038337	CG6125	651	322	22.4457	11.3473	0.0000	-1.0145	down
FBgn0005683	pie	69	21	2.2998	0.7156	0.0006	-1.6678	down
FBgn0032888	CheB38c	1	22	0.2872	3.2464	0.0045	3.4738	up
FBgn0037897	CG5270	1029	659	8.8846	5.8604	0.0002	-0.6434	down
FBgn0038751	CG4770	15	57	0.7429	2.6858	0.0008	1.8518	up
FBgn0015040	Cyp9c1	1225	568	46.1085	21.9182	0.0000	-1.1087	down
FBgn0024987	ssx	2036	4213	37.2023	78.3121	0.0000	1.0474	up
FBgn0052282	Drsl4	2204	453	1838.6400	368.4670	0.0000	-2.2812	down
FBgn0034756	Cyp6d2	95	14	3.7714	0.5672	0.0000	-2.6732	down
FBgn0036680	Cpr73D	800	488	22.8382	14.0631	0.0000	-0.7132	down
FBgn0033373	CG8080	1272	855	45.0741	30.8704	0.0013	-0.5738	down
FBgn0036428	Gbs-70E	4697	3017	105.6127	67.8813	0.0000	-0.6398	down
FBgn0264326	PolE1	145	50	1.2712	0.4506	0.0000	-1.5173	down
FBgn0039151	CG13607	3386	2316	122.1801	88.0084	0.0008	-0.5491	down
FBgn0031942	CG7203	1051	612	159.0654	98.2274	0.0000	-0.7804	down
FBgn0033597	Cpr47Ea	39	2	3.6338	0.1931	0.0000	-3.7048	down
FBgn0038653	Octalpha2R	719	1113	5.5577	10.3857	0.0002	0.6283	up
FBgn0039748	CG15529	186	314	13.2128	25.3030	0.0026	0.7507	up
FBgn0033544	CG7220	5339	8433	231.8693	326.1789	0.0000	0.6580	up
FBgn0026417	Hus1-like	4422	3070	28.9451	20.2723	0.0018	-0.5276	down
FBgn0037936	CG6908	980	2279	51.9151	118.9370	0.0000	1.2153	up
FBgn0264707	RhoGEF3	3552	2346	33.6376	26.2484	0.0001	-0.5995	down
FBgn0002773	Mlc2	9280	14242	279.3711	406.1691	0.0000	0.6166	up
FBgn0025692	Lfg	125	52	11.5801	4.9437	0.0002	-1.2495	down
FBgn0001229	Hsp67Bc	234	127	18.4337	10.1984	0.0010	-0.8775	down
FBgn0039257	tnc	717	460	4.0333	2.6875	0.0008	-0.6405	down
FBgn0039315	CG13658	442	87	21.6381	2.7690	0.0000	-2.3321	down
FBgn0036260	Rh7	446	675	9.2922	12.2774	0.0045	0.5953	up
FBgn0038236	Cyp313a1	5825	8862	251.4380	362.7080	0.0000	0.6039	up
FBgn0032701	CG10341	155	298	5.5036	10.8931	0.0000	0.9369	up
FBgn0034247	CG6484	39	95	1.9483	4.9361	0.0030	1.2601	up
FBgn0033936	Achl	1172	2816	16.3519	39.3484	0.0000	1.2626	up
FBgn0033051	Strica	94	31	2.4270	0.7240	0.0001	-1.5689	down
FBgn0033395	Cyp4p2	89	29	3.6760	1.2048	0.0001	-1.5839	down
FBgn0027259	Kmn1	163	28	10.2514	1.4139	0.0000	-2.4978	down
FBgn0010401	Os-C	68	135	16.5263	33.1941	0.0097	0.9768	up
FBgn0029608	CG3091	1428	865	88.7437	55.5703	0.0000	-0.7239	down
FBgn0040629	CAH5	75	335	5.5916	24.9664	0.0000	2.1419	up
FBgn0035076	Ance-5	2865	1763	89.0852	55.9942	0.0000	-0.7015	down
FBgn0027844	CAH1	1540	986	70.6636	47.4740	0.0000	-0.6440	down
FBgn0038525	CG14329	249	5	18.7036	0.4044	0.0000	-5.3644	down
FBgn0039154	Npc2f	33	3	4.3350	0.3872	0.0002	-3.0648	down
FBgn0033149	CG11060	27	72	0.8298	2.0116	0.0082	1.3788	up
FBgn0265356	tn	2019	2895	27.0233	40.0105	0.0034	0.5183	up
FBgn0005664	Crys	425	82	12.3450	2.4943	0.0000	-2.3599	down
FBgn0051344	CG31344	420	833	23.5374	47.1860	0.0000	0.9848	up
FBgn0050285	CG30285	230	66	46.7816	13.6201	0.0000	-1.7858	down
FBgn0041181	Tep3	958	636	11.9896	7.9824	0.0015	-0.5915	down
FBgn0011591	fng	609	990	22.2121	36.8210	0.0000	0.6987	up
FBgn0033807	AQP	246	525	14.6475	33.3423	0.0000	1.0890	up
FBgn0038172	Adgf-D	811	1525	36.6629	70.6836	0.0000	0.9088	up
FBgn0037999	CG4860	57	166	3.2085	9.9534	0.0000	1.5232	up
FBgn0262029	d	161	42	1.8850	0.5175	0.0000	-1.9130	down
FBgn0039807	CG15546	204	330	7.4863	12.0369	0.0086	0.6897	up
FBgn0051205	CG31205	724	3729	61.2458	293.4567	0.0000	2.3617	up
FBgn0036910	Cyp305a1	1581	2788	68.2123	128.1580	0.0000	0.8166	up
FBgn0036224	Rpt4R	65	138	2.9048	6.2996	0.0018	1.0724	up
FBgn0031865	Nha1	228	514	5.9669	14.3049	0.0000	1.1676	up
FBgn0033443	CG1698	140	54	4.5389	1.7177	0.0000	-1.3583	down
FBgn0267348	LanB2	446	708	4.7285	7.8879	0.0004	0.6641	up
FBgn0020908	Scp1	258	754	22.0500	58.4279	0.0000	1.5419	up
FBgn0039703	CG7829	1638	3229	138.1660	280.8130	0.0000	0.9773	up

FBgn0033268	Obp44a	18238	10444	2493.5837	1462.0496	0.0000	-0.8056	down
FBgn0035164	CG13901	138	239	11.2810	20.0825	0.0063	0.7863	up
FBgn0016122	Acer	4611	2866	127.1850	77.4620	0.0000	-0.6872	down
FBgn0034512	Bbd	6601	9682	741.9890	1115.5062	0.0006	0.5512	up
FBgn0030482	CG1673	498	903	13.9715	24.6334	0.0000	0.8558	up
FBgn0036698	CG7724	828	1201	41.6517	62.6098	0.0063	0.5346	up
FBgn0038407	CG6126	4446	2844	167.7122	113.8938	0.0000	-0.6457	down
FBgn0262035	CG42846	580	375	110.5760	71.7500	0.0029	-0.6290	down
FBgn0029851	CG14445	58	127	2.5891	5.4483	0.0018	1.1150	up
FBgn0004865	Eip78C	977	640	18.3395	11.7301	0.0007	-0.6108	down
FBgn0035777	CG8563	9	49	0.3859	1.6885	0.0001	2.3120	up
FBgn0024989	CG3777	37	92	0.5782	1.6856	0.0027	1.2882	up
FBgn0017558	Pdk	3250	5160	68.6716	103.1768	0.0000	0.6654	up
FBgn0001112	Gld	520	948	11.9511	21.7741	0.0000	0.8637	up
FBgn0011768	Fdh	2695	1701	137.6740	92.4988	0.0000	-0.6649	down
FBgn0038654	CG14298	249	395	28.6496	41.1512	0.0069	0.6621	up
FBgn0038680	Cyp12a5	402	141	16.0285	5.6310	0.0000	-1.5057	down
FBgn0038180	Cht5	93	242	2.9657	7.9917	0.0000	1.3682	up
FBgn0029858	mldr	149	75	6.2006	3.1608	0.0043	-0.9815	down
FBgn0052191	CG32191	404	656	12.7301	21.1596	0.0002	0.6965	up
FBgn0033289	CG2121	258	652	9.4453	25.2241	0.0000	1.3325	up
FBgn0063492	GstE8	251	124	18.9617	8.9297	0.0000	-1.0124	down
FBgn0038632	CG14301	83	32	2.9549	1.0949	0.0033	-1.3472	down
FBgn0037140	SLC22A	385	244	12.9821	8.9016	0.0088	-0.6570	down
FBgn0001325	Kr	48	105	0.8138	1.6437	0.0093	1.1107	up
FBgn0035544	CG15021	1727	1113	95.8452	68.1386	0.0000	-0.6347	down
FBgn0015032	Cyp4c3	107	196	3.7513	7.0312	0.0048	0.8654	up
FBgn0034335	GstE1	1253	757	143.3580	88.6455	0.0000	-0.7276	down
FBgn0035806	PGRP-SD	500	763	60.2226	93.1341	0.0019	0.6073	up
FBgn0032615	CG6012	53	15	4.2485	1.2267	0.0040	-1.7514	down
FBgn0010051	Itpr	3017	1981	23.8465	17.9552	0.0001	-0.6080	down
FBgn0052296	Mrtf	1019	699	8.3688	5.4219	0.0066	-0.5444	down
FBgn0023549	Mct1	604	376	14.1647	9.8931	0.0004	-0.6836	down
FBgn0027932	Akap200	4533	3006	94.8651	65.1654	0.0001	-0.5938	down
FBgn0038312	Zip88E	179	90	7.9086	4.0703	0.0009	-0.9848	down
FBgn0039759	CG9733	47	163	2.1496	7.8584	0.0000	1.7697	up
FBgn0010383	Cyp18a1	305	653	6.8197	16.5572	0.0000	1.0942	up
FBgn0038463	CG3534	856	1456	28.7056	49.2404	0.0000	0.7642	up
FBgn0029170	TwdlT	347	88	14.9097	4.0133	0.0000	-1.9676	down
FBgn0086347	Myo31DF	659	1006	11.0369	16.9691	0.0007	0.6081	up
FBgn0264953	Piezo	2251	1386	16.1645	9.8229	0.0000	-0.7006	down
FBgn0260995	dpr21	879	488	53.2301	24.9308	0.0000	-0.8489	down
FBgn0003356	Jon99Cii	1	34	0.1958	3.6915	0.0000	4.0779	up
FBgn0034920	CG5597	802	1470	84.5241	163.0990	0.0000	0.8719	up
FBgn0050197	CG30197	3895	2710	1076.6800	754.8580	0.0022	-0.5245	down
FBgn0036380	CG8757	177	359	5.9738	11.5366	0.0000	1.0145	up
FBgn0030518	CG11134	421	270	38.3531	25.4560	0.0089	-0.6401	down
FBgn0037519	CG3014	4962	3440	81.9137	54.4847	0.0016	-0.5297	down
FBgn0261792	snRNP-U1-C	67	165	9.0832	22.3527	0.0000	1.2853	up
FBgn0050098	CG30098	60	3	6.8346	0.3335	0.0000	-3.9066	down
FBgn0000473	Cyp6a2	709	1383	27.9007	57.1115	0.0000	0.9615	up
FBgn0031800	CG9497	1945	1169	93.5820	58.1436	0.0000	-0.7353	down
FBgn0039742	CG15528	223	359	17.2992	29.1477	0.0065	0.6830	up
FBgn0035855	CG7366	136	63	2.3293	1.5380	0.0013	-1.0985	down
FBgn0266666	Sem1	377	233	140.7220	81.4631	0.0039	-0.6930	down
FBgn0033603	Cpr47Ef	152	70	5.3371	2.8000	0.0004	-1.1081	down
FBgn0039241	CG11089	2593	3993	83.6162	135.9704	0.0000	0.6213	up
FBgn0002673	twe	71	23	2.0948	0.6608	0.0011	-1.5832	down
FBgn0039777	Jon99Fii	2	52	0.2717	5.1139	0.0000	4.1080	up
FBgn0021795	Tapdelta	1047	1720	126.1530	216.5060	0.0000	0.7142	up
FBgn0038589	CG18598	243	385	149.8120	235.5360	0.0081	0.6602	up
FBgn0028533	CG7953	0	55	0.0707	4.1050	0.0000	5.7034	up
FBgn0036623	Agpat3	2433	1451	115.2249	66.2610	0.0000	-0.7466	down
FBgn0039820	CG15554	12	60	1.3355	6.4492	0.0000	2.2224	up
FBgn0040060	yip7	0	25	0.0000	2.6627	0.0001	4.5988	up
FBgn0028496	CG30116	3693	2504	41.1198	33.7087	0.0004	-0.5617	down
FBgn0035943	CG5653	313	100	17.0624	5.6824	0.0000	-1.6370	down
FBgn0035791	CG8539	289	58	15.9568	3.1683	0.0000	-2.2971	down
FBgn0050101	Vajk4	68	159	3.4045	8.2948	0.0000	1.2112	up
FBgn0002577	m	5	32	0.1136	0.6268	0.0058	2.4435	up
FBgn0050295	Ipk1	865	1483	20.4332	35.8906	0.0000	0.7757	up
FBgn0261575	tobi	1541	2294	53.7187	83.3145	0.0004	0.5723	up
FBgn0053143	CG33143	3019	1436	38.8040	15.2777	0.0000	-1.0728	down
FBgn0037534	ELOVL	1482	2306	53.2454	80.5675	0.0000	0.6361	up
FBgn0003719	tld	45	144	0.8787	2.8628	0.0000	1.6534	up
FBgn0028841	jhamt	6	41	0.5097	3.1988	0.0002	2.5708	up
FBgn0051343	CG31343	4	86	0.1122	2.0179	0.0000	4.0994	up
FBgn0038214	CG9616	46	117	7.1973	4.7736	0.0001	1.3253	up
FBgn0021738	Crg-1	197	383	10.6979	18.1603	0.0000	0.9540	up
FBgn0037126	CG14567	204	340	27.3002	46.5775	0.0025	0.7326	up
FBgn0038467	AdSL	750	1181	20.6977	33.1968	0.0001	0.6530	up
FBgn0031249	CG11911	3	29	0.3085	2.4357	0.0021	2.8823	up
FBgn0038983	CG5326	689	1118	32.1475	53.8108	0.0000	0.6961	up
FBgn0039049	CG6726	119	212	5.4807	10.0588	0.0061	0.8261	up
FBgn0020270	mre11	768	446	27.2772	16.1619	0.0000	-0.7839	down
FBgn0036004	Jarid2	2078	1434	14.5474	10.1186	0.0023	-0.5362	down
FBgn0262366	CG43064	101	37	3.2020	1.0828	0.0002	-1.4240	down
FBgn0024836	stan	934	577	4.5739	2.9574	0.0000	-0.6952	down
FBgn0031971	Sirup	1245	786	169.0980	103.4570	0.0000	-0.6642	down
FBgn0037683	CG18473	17	292	1.0164	17.1661	0.0000	4.0179	up
FBgn0032068	LManV	0	156	0.0000	3.1675	0.0000	7.1894	up
FBgn0005660	Ets21C	18	88	0.3029	2.5877	0.0000	2.2220	up

FBgn0052251	Claspin	239	29	2.7315	0.3367	0.0000	-2.9981	down
FBgn0052823	Sdic3	114	0	3.3475	0.0000	0.0000	-6.7419	down
FBgn0033215	Dgat2	50	168	3.3216	11.4124	0.0000	1.7256	up
FBgn0029657	CG12535	118	263	1.2269	2.2652	0.0000	1.1477	up
FBgn0259834	out	308	592	7.2952	14.4064	0.0000	0.9389	up
FBgn0034295	CG10911	1	54	0.1415	4.0684	0.0000	4.7288	up
FBgn0052475	mthl8	807	1411	15.7023	29.4253	0.0000	0.8039	up
FBgn0038098	CG7381	380	200	8.0197	4.4115	0.0000	-0.9237	down
FBgn0001083	fw	318	526	4.6564	7.9385	0.0003	0.7228	up
FBgn0038816	Lrrk	1257	877	10.3186	7.2876	0.0095	-0.5201	down
FBgn0031775	CG9150	183	306	17.7164	30.8729	0.0043	0.7370	up
FBgn0051207	CG31207	645	1086	62.7862	103.5566	0.0000	0.7493	up
FBgn0035239	CG18170	133	39	1.6103	0.6620	0.0000	-1.7436	down
FBgn0022355	Tsf1	91529	58874	2922.0622	2018.2425	0.0000	-0.6379	down
FBgn0035189	CG9119	892	1334	52.7675	83.9815	0.0010	0.5787	up
FBgn0262003	CG42821	207	100	54.2216	13.7726	0.0001	-1.0430	down
FBgn0038074	Gnmt	656	4138	52.3340	336.3220	0.0000	2.6538	up
FBgn0044812	TotC	3399	1479	702.3930	304.0840	0.0000	-1.2012	down
FBgn0011281	Obp83a	119	341	12.8107	38.4750	0.0000	1.5090	up
FBgn0036935	CG14186	2453	3652	44.4451	67.7727	0.0003	0.5726	up
FBgn0035873	CG13670	410	218	35.8016	20.0083	0.0000	-0.9093	down
FBgn0053971	Ir62a	162	76	6.8447	3.2459	0.0003	-1.0825	down
FBgn0038301	CG6654	616	2052	17.9631	61.6944	0.0000	1.7329	up
FBgn0000109	Aprt	134	236	12.2732	22.0815	0.0041	0.8102	up
FBgn0260429	CG42524	22	81	0.5840	1.9728	0.0000	1.8293	up
FBgn0041087	wun2	233	131	9.0969	5.1566	0.0031	-0.8269	down
FBgn0035607	CG4835	51	0	0.9114	0.0000	0.0000	-5.5980	down
FBgn0037547	CG7910	7	77	0.2942	2.8987	0.0000	3.2720	up
FBgn0004244	Rdl	5779	8262	37.9170	48.1176	0.0033	0.5142	up
FBgn0058191	CG40191	896	1391	43.6331	60.2464	0.0001	0.6326	up
FBgn0052523	CG32523	676	1263	61.1685	118.1530	0.0000	0.8994	up
FBgn0040502	CG8343	17524	45221	2421.9900	6484.0600	0.0000	1.3663	up
FBgn0262717	Skeletor	364	806	9.0291	14.5237	0.0000	1.1432	up
FBgn0039024	Nepl15	2364	1610	70.4406	49.0757	0.0009	-0.5552	down
FBgn0023535	arg	12	57	0.8460	3.9488	0.0001	2.1498	up
FBgn0042131	CG18808	5549	3771	23.2258	16.1377	0.0004	-0.5585	down
FBgn0051233	CG31233	0	43	0.0000	0.9796	0.0000	5.3560	up
FBgn0051973	Cda5	1179	629	13.2729	7.7352	0.0000	-0.9066	down
FBgn0033593	Listericin	1324	1887	394.4940	579.2110	0.0073	0.5095	up
FBgn0039487	gb	279	562	7.9834	17.1759	0.0000	1.0062	up
FBgn0039214	puf	1694	1147	7.6326	5.2934	0.0011	-0.5635	down
FBgn0038038	Sccpdh2	1369	1965	65.5575	98.4636	0.0047	0.5197	up
FBgn0036790	AstC-R1	169	515	4.6408	13.0752	0.0000	1.6001	up
FBgn0036139	CG6216	24	0	1.5453	0.0000	0.0001	-4.5436	down
FBgn0052212	CG32212	40	7	5.8863	1.0524	0.0010	-2.3479	down
FBgn0002565	Lsp2	9192	18587	289.3705	618.0670	0.0000	1.0144	up
FBgn0033542	CAH13	105	37	4.0727	1.4637	0.0000	-1.4795	down
FBgn0004197	Ser	12	54	0.1443	0.6294	0.0002	2.0733	up
FBgn0032821	CdGAPr	1908	1183	16.5154	10.0558	0.0000	-0.6905	down
FBgn0038676	CG6026	32	2	0.3684	0.0242	0.0001	-3.4279	down
FBgn0031653	Jon25Biii	0	60	0.0000	6.8430	0.0000	5.8266	up
FBgn0036549	CG10516	1428	556	78.1849	31.2305	0.0000	-1.3605	down
FBgn0034647	pirk	79	211	4.1349	11.0073	0.0000	1.4038	up
FBgn0052368	CG32368	59	18	29.6660	8.8561	0.0033	-1.6563	down
FBgn0035915	S-Lap1	114	44	3.6850	1.4704	0.0001	-1.3535	down
FBgn0051380	CG31380	1	36	0.1151	2.0741	0.0000	4.1579	up
FBgn0020277	lush	67	145	5.8118	12.5659	0.0007	1.1002	up
FBgn0038201	PK1-R	86	227	2.8139	8.1839	0.0000	1.3878	up
FBgn0036106	CG6409	10563	14973	689.6004	1051.6252	0.0076	0.5020	up
FBgn0051216	Naam	149	264	5.6015	10.1384	0.0016	0.8194	up
FBgn0037344	CG2926	2772	1951	18.7875	13.3048	0.0053	-0.5078	down
FBgn0014455	Ahcy	1518	2239	65.2819	100.7956	0.0008	0.5590	up
FBgn0032741	Sidpn	866	1296	23.9456	37.0975	0.0010	0.5797	up
FBgn0037265	spartin	873	436	28.2619	14.3977	0.0000	-1.0012	down
FBgn0037167	CG11425	163	52	14.2930	4.8259	0.0000	-1.6296	down
FBgn0031490	CG17264	199	28	4.9084	0.7415	0.0000	-2.7840	down
FBgn0025595	AkhR	1552	635	56.9245	23.6360	0.0000	-1.2892	down
FBgn0034515	CG13428	440	947	129.6303	278.8758	0.0000	1.1026	up
FBgn0028542	NimB4	270	440	14.2912	24.2335	0.0014	0.7010	up
FBgn0016685	Nlp	1083	673	95.9684	59.7882	0.0000	-0.6868	down
FBgn0037222	CG14642	9	78	0.5082	4.1024	0.0000	2.9711	up
FBgn0032381	Mal-B1	164	81	6.6652	3.3305	0.0012	-1.0094	down
FBgn0028396	TotA	6737	4045	1379.7700	806.8670	0.0000	-0.7372	down
FBgn0039629	CG11842	532	237	37.9756	17.3585	0.0000	-1.1643	down
FBgn0036262	CG6910	9974	18494	483.5548	872.0271	0.0000	0.8894	up
FBgn0032494	CG5945	5873	9409	428.5591	705.8371	0.0000	0.6785	up
FBgn0051075	CG31075	1717	1104	67.9112	46.2427	0.0000	-0.6380	down
FBgn0031791	AANATL2	105	39	11.5173	4.1727	0.0001	-1.4056	down
FBgn0001124	Got1	4857	7378	201.3794	315.1316	0.0000	0.6017	up
FBgn0004654	Pgd	3053	4504	123.4122	194.8188	0.0004	0.5595	up
FBgn0029831	CG5966	1101	1957	28.4548	51.5419	0.0000	0.8279	up
FBgn0026403	Ndg	435	169	5.8832	2.3886	0.0000	-1.3597	down
FBgn0038387	blp	210	410	18.0705	35.2945	0.0000	0.9603	up
FBgn0037788	CAH7	1315	763	61.4051	37.8309	0.0000	-0.7858	down
FBgn0000078	Amy-d	762	467	31.2957	20.6601	0.0001	-0.7064	down
FBgn0041249	Gr22f	0	18	0.0734	1.3374	0.0062	4.1478	up
FBgn0028394	CG17834	217	352	9.0385	15.5423	0.0055	0.6938	up
FBgn0014863	Mlp84B	3495	5988	95.1945	163.3246	0.0000	0.7753	up
FBgn0051407	CG31407	4810	8782	28.2399	53.6870	0.0000	0.8670	up
FBgn0015075	Ddx1	452	292	11.7992	7.8983	0.0085	-0.6298	down
FBgn0039239	CG13641	1	27	0.4927	7.1434	0.0002	3.7567	up
FBgn0002736	mago	147	288	10.7453	20.2037	0.0000	0.9638	up

FBgn0026602	Adk3	733	1777	20.5542	43.1709	0.0000	1.2750	up
FBgn0032835	CG16772	3475	2344	271.0610	194.0058	0.0003	-0.5692	down
FBgn0259716	Nepl6	609	401	20.3909	13.6371	0.0053	-0.6029	down
FBgn0035227	Iml1	4769	1762	61.9610	23.1910	0.0000	-1.4373	down
FBgn0027657	glob1	4689	2955	430.4122	269.3227	0.0000	-0.6673	down
FBgn0058263	MFS17	998	666	45.5711	28.5540	0.0018	-0.5841	down
FBgn0040207	kat80	1002	560	18.9028	10.6938	0.0000	-0.8395	down
FBgn0033464	CG1441	1018	2221	39.8784	90.6059	0.0000	1.1233	up
FBgn0037046	CG10581	61	134	10.2948	22.4560	0.0010	1.1203	up
FBgn0032698	CG10336	8	75	0.5341	4.6303	0.0000	3.0661	up
FBgn0040491	Buffy	273	451	22.2326	37.7991	0.0007	0.7207	up
FBgn0035434	Drsl5	2375	990	1278.8900	522.7750	0.0000	-1.2629	down
FBgn0001168	h	883	1401	26.0051	40.6017	0.0000	0.6640	up
FBgn0037773	CG5359	48	108	2.7275	6.6088	0.0043	1.1509	up
FBgn0037410	Osi2	221	1374	10.5406	70.1504	0.0000	2.6290	up
FBgn0030993	Mec2	196	418	11.4061	26.6136	0.0000	1.0871	up
FBgn0001137	grk	402	674	16.5433	27.6453	0.0000	0.7427	up
FBgn0038020	GstD9	754	477	86.6483	55.5647	0.0003	-0.6607	down
FBgn0014019	Rh5	10577	6630	669.3242	433.9447	0.0000	-0.6751	down
FBgn0001256	ImpL1	61	12	3.9083	0.8152	0.0000	-2.2484	down
FBgn0000045	Act79B	1294	2309	64.7631	122.8125	0.0000	0.8336	up
FBgn0067312	CheB38a	1	23	0.4185	5.0348	0.0024	3.5350	up
FBgn0033153	Gadd45	146	350	6.8410	16.7921	0.0000	1.2539	up
FBgn0083973	dunk	0	29	0.0836	2.5863	0.0000	4.8047	up
FBgn0010423	TpnC47D	0	21	0.0000	2.8881	0.0008	4.3585	up
FBgn0029521	Or1a	9	44	0.7002	3.0535	0.0012	2.1602	up
FBgn0032891	Oseg5	51	15	1.2367	0.3756	0.0088	-1.6970	down
FBgn0086704	stops	4254	2668	126.9119	83.9774	0.0000	-0.6742	down
FBgn0085195	CG34166	932	2232	377.4976	841.4743	0.0000	1.2576	up
FBgn0034330	BomS4	70	0	152.7930	0.0000	0.0000	-6.0467	down
FBgn0016797	fz2	2834	1875	33.9935	21.7546	0.0001	-0.5970	down
FBgn0024811	Crk	1935	1313	78.4979	52.0509	0.0009	-0.5604	down
FBgn0004181	Ebp	4	45	0.3475	2.4315	0.0000	3.1812	up
FBgn0053494	CG33494	815	356	91.4545	37.0090	0.0000	-1.1938	down
FBgn0034276	Sardh	199	401	4.6674	9.8152	0.0000	1.0056	up
FBgn0031489	CG17224	968	505	55.6752	30.9236	0.0000	-0.9386	down
FBgn0039800	Npc2g	8520	3450	1097.5120	385.5162	0.0000	-1.3053	down
FBgn0029588	CR14798	22	69	2.0083	6.4400	0.0012	1.6012	up
FBgn0015037	Cyp4p1	1109	2862	42.0412	106.3470	0.0000	1.3656	up
FBgn0039518	CG13978	2	39	0.0706	0.8848	0.0000	3.7027	up
FBgn0034117	CG7997	3208	2212	142.1044	101.2441	0.0014	-0.5374	down
FBgn0053926	CG33926	641	2953	69.2200	338.2795	0.0000	2.2006	up
FBgn0041709	yellow-g	62	21	3.5318	1.4720	0.0076	-1.5159	down
FBgn0037164	CG11438	178	312	4.5958	8.2424	0.0007	0.8046	up
FBgn0051198	CG31198	0	96	0.0213	2.1519	0.0000	6.4952	up
FBgn0085334	CG34305	14	81	18.0699	94.0310	0.0000	2.4434	up
FBgn0038821	CG17267	130	19	6.7295	1.0045	0.0000	-2.7082	down
FBgn0030439	CG12716	103	3	4.4398	0.1708	0.0000	-4.6756	down
FBgn0032187	CG4839	68	23	0.7554	0.3195	0.0031	-1.5219	down
FBgn0086677	jeb	2881	4118	17.4867	23.2713	0.0034	0.5139	up
FBgn0033728	Cpr49Ae	776	503	45.7538	30.3130	0.0010	-0.6258	down
FBgn0034331	BomBc2	3351	1342	771.9170	359.2087	0.0000	-1.3209	down
FBgn0036015	CG3088	16779	31137	1900.1300	3681.2500	0.0000	0.8906	up
FBgn0034328	BomBc1	1036	1	323.4800	0.3227	0.0000	-8.9657	down
FBgn0041180	Tep4	13072	5375	171.4275	72.5376	0.0000	-1.2833	down
FBgn0039152	Root	881	211	6.7997	1.7238	0.0000	-2.0577	down
FBgn0037797	CG12420	0	26	0.0000	2.9493	0.0000	4.6531	up
FBgn0250815	Jon65Aiv	1	21	0.1733	2.0177	0.0084	3.4099	up
FBgn0034440	CG10073	31	5	0.8745	0.1410	0.0095	-2.4016	down
FBgn0033052	SCAP	1406	2674	17.0396	32.2499	0.0000	0.9255	up
FBgn0037443	Dmtn	4068	2793	76.0316	55.9408	0.0009	-0.5437	down
FBgn0050489	Cyp12d1-p	57	10	2.2363	0.4379	0.0000	-2.3918	down
FBgn0026755	Ugt37B1	52	3	2.1655	0.1314	0.0000	-3.7041	down
FBgn0015527	peng	1011	519	27.3757	13.4083	0.0000	-0.9619	down
FBgn0047095	CG33939	17	60	3.6420	11.6974	0.0011	1.7553	up
FBgn0043791	phu	4094	237	151.8800	9.2372	0.0000	-4.1057	down
FBgn0034687	CG11475	979	533	37.4009	21.5457	0.0000	-0.8772	down
FBgn0040828	CG13306	2	43	0.6616	9.6501	0.0000	3.8399	up
FBgn0262574	CG43114	126	51	263.8724	97.6733	0.0001	-1.2883	down
FBgn0259229	CG42329	502	202	15.0442	7.9261	0.0000	-1.3101	down
FBgn0034802	CNBP	1638	228	46.6269	5.7110	0.0000	-2.8403	down
FBgn0034394	CG15096	2930	4159	121.8024	184.0544	0.0052	0.5038	up
FBgn0050428	CG30428	162	325	4.0509	7.0538	0.0000	0.9983	up
FBgn0029823	Shmt	2953	5291	120.5957	228.6004	0.0000	0.8398	up
FBgn0040682	CG14664	26	2	1.5030	0.1068	0.0024	-3.1391	down
FBgn0264343	CG43799	1	36	0.2015	3.2087	0.0000	4.1579	up
FBgn0028534	CG7916	0	56	0.0884	5.2643	0.0000	5.7289	up
FBgn0042201	Nplp3	7220	4900	1607.4600	1143.3700	0.0004	-0.5605	down
FBgn0050431	CG30431	1014	641	35.2242	23.6190	0.0001	-0.6621	down
FBgn0035982	CG4461	677	361	63.3133	34.6832	0.0000	-0.9065	down
FBgn0051664	CG31664	271	104	9.2643	3.5992	0.0000	-1.3739	down
FBgn0038017	CG4115	207	104	14.0771	7.3126	0.0002	-0.9870	down
FBgn0030590	CG9518	52	8	0.8963	0.1361	0.0000	-2.5492	down
FBgn0031701	TotM	208	69	45.7751	15.3988	0.0000	-1.5784	down
FBgn0027348	bgm	2372	1055	57.2117	25.0712	0.0000	-1.1694	down
FBgn0000052	Pfas	1982	3185	28.2030	47.6571	0.0000	0.6827	up
FBgn0036359	CG14105	83	228	11.5027	30.9083	0.0000	1.4447	up
FBgn0085521	CG40813	0	22	0.0000	3.4809	0.0004	4.4224	up
FBgn0039114	Lsd-1	9628	3543	474.9345	180.1669	0.0000	-1.4433	down
FBgn0263256	CG43394	436	653	9.7673	15.2657	0.0080	0.5802	up
FBgn0037288	CG14661	3111	4871	215.8124	332.7273	0.0000	0.6453	up
FBgn0035921	CG13305	808	264	27.1793	10.5932	0.0000	-1.6112	down

FBgn0033665	Zip48C	242	402	8.9633	15.1636	0.0010	0.7283	up
FBgn0034071	CG8405	2607	1730	47.7011	31.1526	0.0001	-0.5927	down
FBgn0032613	Nepl9	274	435	9.0598	14.3199	0.0041	0.6634	up
FBgn0036876	CG9451	885	601	43.5850	29.4478	0.0060	-0.5588	down
FBgn0263974	qin	85	18	0.8458	0.1789	0.0000	-2.1752	down
FBgn0038063	Octbeta2R	1377	2172	15.7442	21.9398	0.0000	0.6557	up
FBgn0031558	CG16704	297	179	221.3586	124.0402	0.0061	-0.7284	down
FBgn0259998	CG17571	1	40	0.1714	3.7105	0.0000	4.3057	up
FBgn0261714	Cpn	4207	6449	117.3477	185.5137	0.0000	0.6148	up
FBgn0038295	Gyc88E	679	418	9.4889	6.1475	0.0001	-0.6998	down
FBgn0037548	CG7900	140	419	4.7352	14.2094	0.0000	1.5728	up
Trans_newGene_22	-	105	204	2.9633	6.8697	0.0006	0.9497	up
Trans_newGene_37	-	350	4	2.5222	0.0262	0.0000	-6.1132	down
Trans_newGene_85	-	15	111	0.3973	2.4071	0.0000	2.8002	up
Trans_newGene_87	-	311	1112	2.4693	8.4437	0.0000	1.8332	up
Trans_newGene_219	-	119	228	13.4217	20.2327	0.0004	0.9305	up
Trans_newGene_313	-	0	41	0.0000	0.6688	0.0000	5.2890	up
Trans_newGene_363	-	40	3	2.2471	0.1665	0.0000	-3.3343	down
Trans_newGene_378	-	621	175	6.1607	2.4058	0.0000	-1.8222	down
Trans_newGene_383	-	60	157	0.7289	1.7315	0.0000	1.3706	up
Trans_newGene_384	-	106	299	1.1714	3.0878	0.0000	1.4854	up
Trans_newGene_418	-	4	125	0.2708	7.0878	0.0000	4.6334	up
Trans_newGene_420	-	1143	1832	38.2904	61.8007	0.0000	0.6787	up

#ID	gene_name	w^{1118}_ZT14_Count	$14\text{-}3\text{-}3\varepsilon^{EP3578}$_ZT14_Count	w^{1118}_ZT14_FPKM	$14\text{-}3\text{-}3\varepsilon^{EP3578}$_ZT14_FPKM	FDR	log2FC	regulated
FBgn0259795	loopin-1	39	0	1.4914	0.0000	0.0000	-5.1421	down
FBgn0067903	IM18	103	19	195.9760	37.1114	0.0000	-2.3877	down
FBgn0011770	Gip	627	1092	54.9950	95.0515	0.0000	0.7812	up
FBgn0039325	CG10560	458	1417	21.7099	68.0286	0.0000	1.6089	up
FBgn0039073	CG4408	213	732	9.4242	31.9554	0.0000	1.7574	up
FBgn0040465	Dlip3	424	236	21.9111	12.1004	0.0000	-0.8603	down
FBgn0036105	Blos4	118	33	11.3770	3.2663	0.0000	-1.8207	down
FBgn0038739	CG4686	256	445	25.8328	44.0089	0.0003	0.7769	up
FBgn0263200	Galt	862	1862	42.7608	91.1071	0.0000	1.0920	up
FBgn0037713	CG16790	325	712	21.5303	50.1723	0.0000	1.1106	up
FBgn0031859	CG17377	25	2	3.4138	0.2640	0.0045	-3.0703	down
FBgn0032402	PLCXD	787	1310	25.9776	41.2104	0.0000	0.7162	up
FBgn0259145	CG42260	1531	672	11.4111	5.1019	0.0000	-1.2047	down
FBgn0038840	Grik	549	307	11.4538	6.2528	0.0000	-0.8543	down
FBgn0037396	CG11459	151	48	8.1710	2.6335	0.0000	-1.6482	down
FBgn0039543	CROT	798	529	24.9529	16.4057	0.0029	-0.6102	down
FBgn0016013	Faa	340	749	17.3866	38.0724	0.0000	1.1187	up
FBgn0020299	stumps	675	1112	10.0241	15.7224	0.0000	0.7011	up
FBgn0032264	Lip4	660	364	29.6569	14.8966	0.0000	-0.8746	down
FBgn0037687	CG8132	205	412	16.5033	32.7102	0.0000	0.9849	up
FBgn0013279	Hsp70Bc	46	3	1.2176	0.0798	0.0000	-3.5234	down
FBgn0038986	sit	106	38	4.7664	1.7288	0.0001	-1.4705	down
FBgn0034480	CG16898	1029	370	56.8543	20.7183	0.0000	-1.4909	down
FBgn0030313	Reepl1	54	0	5.3287	0.0000	0.0000	-5.6002	down
FBgn0013772	Cyp6a8	857	560	30.8243	20.1283	0.0012	-0.6309	down
FBgn0037537	CG2767	2432	3958	127.1150	204.5540	0.0000	0.6842	up
FBgn0020621	Pkn	7553	11140	97.5140	172.4393	0.0035	0.5424	up
FBgn0031418	CG3609	1056	1603	71.6848	106.6020	0.0014	0.5835	up
FBgn0001208	Hn	4045	6449	199.5941	314.0726	0.0000	0.6547	up
FBgn0000261	Cat	6283	10157	212.5950	335.4010	0.0000	0.6747	up
FBgn0052037	CG32037	93	32	3.8781	1.3167	0.0001	-1.5238	down
FBgn0003651	svp	1007	1479	17.2341	25.0021	0.0087	0.5359	up
FBgn0050499	Rpe	329	562	29.2734	47.9432	0.0001	0.7523	up
FBgn0027070	Ugt36E1	346	544	14.9964	23.5141	0.0059	0.6330	up
FBgn0039686	CG15506	37	114	1.9221	5.1414	0.0000	1.5761	up
FBgn0029837	Tsp5D	702	1046	27.4839	40.8073	0.0083	0.5565	up
FBgn0031080	CG12655	342	29	63.7377	5.4512	0.0000	-3.5266	down
FBgn0031545	CG3213	25	0	0.7143	0.0000	0.0001	-4.5232	down
FBgn0033499	CG12914	401	228	10.8950	6.1786	0.0001	-0.8295	down
FBgn0031097	obst-A	219	107	9.9948	4.2144	0.0001	-1.0435	down
FBgn0003390	shf	236	614	9.5608	24.6835	0.0000	1.3571	up
FBgn0050263	stum	68	243	1.7931	3.4556	0.0000	1.8021	up
FBgn0038665	euc	540	882	156.4780	255.6885	0.0001	0.6885	up
FBgn0267398	Yeti	938	1481	78.4531	132.4385	0.0002	0.6402	up
FBgn0036824	CG3902	1976	3893	90.7644	173.9690	0.0000	0.9598	up
FBgn0039081	Irk2	1673	2445	59.9678	87.6879	0.0064	0.5290	up
FBgn0259992	CG42489	20	64	11.0525	34.4889	0.0029	1.6059	up
FBgn0027552	CG10863	3648	6053	258.0830	426.0060	0.0000	0.7122	up
FBgn0086558	Ubi-p5E	9888	3198	295.7020	91.1233	0.0000	-1.6463	down
FBgn0037746	CG8478	56	18	1.7480	0.5635	0.0092	-1.5951	down
FBgn0023178	Pdf	1640	1007	236.0200	143.5690	0.0000	-0.7211	down
FBgn0002868	MtnA	6307	9350	4939.5980	7680.2990	0.0023	0.5498	up
FBgn0051955	CG31955	36	5	3.0325	0.4915	0.0007	-2.6121	down
FBgn0038339	CG6118	0	25	0.0000	0.4107	0.0001	4.5059	up
FBgn0028970	betaggt-II	597	906	35.3259	51.9851	0.0050	0.5827	up
FBgn0034356	Pepck2	83	473	2.0358	12.0053	0.0000	2.4764	up
FBgn0004513	Mdr65	1673	1170	26.4947	18.4925	0.0078	-0.5336	down
FBgn0040609	CG3348	146	59	30.2725	12.3646	0.0000	-1.3086	down
FBgn0037895	CG6723	274	459	11.0283	18.0328	0.0010	0.7238	up
FBgn0032647	CG15143	11	57	0.1710	0.8747	0.0000	2.2423	up
FBgn0038142	CheA87a	298	61	46.7697	9.7385	0.0000	-2.2851	down
FBgn0010387	Acbp2	707	1086	430.3374	662.1674	0.0017	0.6003	up
FBgn0040323	GNBP1	421	250	15.6783	9.2660	0.0004	-0.7673	down
FBgn0031850	Tsp	2872	4376	95.2733	143.5138	0.0004	0.5892	up
FBgn0032350	CG6287	3309	5449	197.5710	317.7690	0.0000	0.7013	up
FBgn0036619	Cpr72Ec	775	2867	30.7999	112.4460	0.0000	1.8676	up
FBgn0038516	P5cr-2	114	247	9.7308	20.8395	0.0000	1.0897	up
FBgn0035817	CG7409	1305	775	178.5540	105.4550	0.0000	-0.7690	down
FBgn0033942	Cpr51A	114	4	18.5230	0.6559	0.0000	-4.5005	down
FBgn0035831	Pop4	132	58	13.0782	5.7802	0.0004	-1.1886	down
FBgn0030607	dob	341	749	12.5443	27.2686	0.0000	1.1145	up
FBgn0028482	bdl	5107	8525	151.1162	244.8090	0.0000	0.7210	up
FBgn0023477	Taldo	2812	5112	203.3960	359.8030	0.0000	0.8439	up
FBgn0038795	CG4335	226	506	14.4961	32.3733	0.0000	1.1407	up
FBgn0010288	Uch	497	825	36.7245	60.2352	0.0001	0.7117	up
FBgn0042175	CG18858	325	563	12.2329	21.2140	0.0001	0.7725	up
FBgn0035550	CG11349	23	1	0.8836	0.0385	0.0026	-3.5063	down
FBgn0027073	Ugt49B1	410	710	16.5027	28.5042	0.0000	0.7724	up
FBgn0023530	CG3740	852	1354	39.5604	49.2075	0.0002	0.6495	up
FBgn0032281	CG17107	84	242	39.5378	112.6030	0.0000	1.4959	up
FBgn0264090	CG43759	321	175	2.9506	1.6353	0.0001	-0.8890	down
FBgn0033158	CG12164	9	43	0.4561	2.0773	0.0026	2.1047	up
FBgn0039685	Obp99b	8865	4818	2055.7807	1106.1007	0.0000	-0.8977	down
FBgn0035942	ValRS-m	353	218	7.3925	4.5272	0.0051	-0.7105	down
FBgn0262150	CG42876	63	5	2.9502	0.3244	0.0000	-3.4004	down
FBgn0033366	Ance-4	586	932	23.1418	36.0278	0.0005	0.6503	up
FBgn0039722	Capa	1644	1023	239.0550	147.4600	0.0000	-0.7019	down
FBgn0038056	CG5961	513	247	23.3252	12.2994	0.0000	-1.0691	down
FBgn0051453	pch2	75	158	3.0297	6.1847	0.0012	1.0456	up
FBgn0005391	Yp2	19	105	0.8942	4.8270	0.0000	2.3801	up
FBgn0033020	COX4L	2	26	0.4412	1.5322	0.0033	3.0956	up

FBgn0038292	CG3987	16	97	0.8034	4.8080	0.0000	2.4998	up
FBgn0035941	CG13313	65	6	3.8923	0.3579	0.0000	-3.2274	down
FBgn0013813	Dhc98D	58	423	0.2522	1.7876	0.0000	2.8243	up
FBgn0029950	CG9657	368	584	11.2617	18.4633	0.0031	0.6465	up
FBgn0030060	CG2004	787	1170	34.5779	51.4720	0.0073	0.5533	up
FBgn0260874	Ir76a	207	389	8.6165	16.2078	0.0000	0.8884	up
FBgn0034329	BomS1	860	452	477.3800	252.7710	0.0000	-0.9444	down
FBgn0031601	Dim1	77	166	15.7088	33.8293	0.0004	1.0790	up
FBgn0039299	CG11854	27	1	2.0703	0.0752	0.0002	-3.7275	down
FBgn0036547	CG17032	626	332	36.3616	18.2741	0.0000	-0.9307	down
FBgn0044047	Ilp6	449	766	21.5386	30.9751	0.0000	0.7510	up
FBgn0010225	Gel	8622	12845	187.0559	273.6998	0.0020	0.5569	up
FBgn0042178	Apl	65	220	1.1719	3.9145	0.0000	1.7234	up
FBgn0038682	CG5835	274	577	16.0937	33.2289	0.0000	1.0532	up
FBgn0003082	phr	65	653	2.5429	25.2961	0.0000	3.2880	up
FBgn0004629	Cys	4025	8089	1332.8500	2683.2700	0.0000	0.9886	up
FBgn0039782	CG15539	55	11	2.3569	0.5033	0.0000	-2.2260	down
FBgn0031012	CG8051	190	351	5.8517	10.7946	0.0001	0.8635	up
FBgn0038002	CG12256	43	8	4.0314	0.7544	0.0006	-2.2881	down
FBgn0004403	RpS14a	11364	17340	1788.1518	2825.9433	0.0005	0.5915	up
FBgn0036290	CG10638	794	1340	52.1618	86.1469	0.0000	0.7361	up
FBgn0022774	Oat	509	877	26.4098	44.3144	0.0000	0.7655	up
FBgn0030157	CG1468	893	1477	107.1630	176.7600	0.0000	0.7071	up
FBgn0035766	eco	504	1563	8.6792	26.9935	0.0000	1.6125	up
FBgn0031563	CG10031	432	678	87.9893	138.8130	0.0028	0.6308	up
FBgn0036774	mRpS26	326	199	38.4741	22.9815	0.0052	-0.7269	down
FBgn0032652	CG6870	2785	4378	265.5395	410.5554	0.0000	0.6343	up
FBgn0033065	Cyp6w1	3614	7098	131.0257	257.9996	0.0000	0.9555	up
FBgn0010403	Obp83b	146	35	37.3186	9.0868	0.0000	-2.0432	down
FBgn0016075	vkg	2344	3564	23.7690	35.2011	0.0005	0.5862	up
FBgn0002865	Mst98Ca	39	6	2.7117	0.4083	0.0005	-2.5068	down
FBgn0000206	boss	3597	5447	87.3645	130.8242	0.0006	0.5804	up
FBgn0050419	CG30419	671	434	9.3917	6.1553	0.0017	-0.6454	down
FBgn0015035	Cyp4e3	634	271	24.0019	10.3252	0.0000	-1.2408	down
FBgn0053138	AGBE	1566	2638	47.8313	78.6867	0.0000	0.7338	up
FBgn0037607	CG8036	4624	10393	149.5930	326.0811	0.0000	1.1501	up
FBgn0036560	CG5895	692	1258	30.4947	55.2050	0.0000	0.8431	up
FBgn0000500	Dsk	2121	1020	456.8620	220.5840	0.0000	-1.0735	down
FBgn0085419	Rgk2	1652	980	21.7193	12.8054	0.0000	-0.7708	down
FBgn0037304	CG1113	86	15	3.0410	0.5566	0.0000	-2.4498	down
FBgn0040349	CG3699	409	2153	45.8751	234.6660	0.0000	2.3748	up
FBgn0003076	Pgm1	3165	4670	122.0470	176.7560	0.0028	0.5429	up
FBgn0036679	CG13022	51	15	8.9785	2.6608	0.0081	-1.7088	down
FBgn0034564	CG9344	20	83	7.0270	29.3896	0.0000	1.9751	up
FBgn0045770	S-Lap3	49	2	1.3901	0.0692	0.0000	-4.0101	down
FBgn0028983	Spn55B	442	173	25.1103	9.6692	0.0000	-1.3655	down
FBgn0065108	ppk16	36	6	0.7064	0.1544	0.0023	-2.3948	down
FBgn0011282	Obp84a	22	0	4.0161	0.0000	0.0005	-4.3474	down
FBgn0034468	Obp56a	72	186	15.2292	39.5714	0.0000	1.3374	up
FBgn0038160	CG9759	537	292	36.3032	19.6293	0.0000	-0.8945	down
FBgn0039697	CG7834	2938	4633	225.1290	343.8853	0.0000	0.6388	up
FBgn0033760	CG8785	224	98	8.3790	3.6131	0.0000	-1.2012	down
FBgn0010591	Sply	931	1375	33.1968	48.0856	0.0075	0.5439	up
FBgn0031360	CG31937	879	1384	59.9439	92.5311	0.0002	0.6361	up
FBgn0023507	D2hgdh	2420	7079	82.3466	232.7558	0.0000	1.5300	up
FBgn0010019	Cyp4g1	186	9	5.7440	0.2800	0.0000	-4.2225	down
FBgn0038981	CG5346	683	1182	22.9158	39.5602	0.0000	0.7721	up
FBgn0024289	Sodh-1	1636	4433	111.6033	299.9469	0.0000	1.4194	up
FBgn0040074	retinin	14653	23346	1742.9200	2712.4900	0.0000	0.6538	up
FBgn0082831	pps	2111	3139	17.8809	26.3936	0.0021	0.5540	up
FBgn0046294	CG12699	31	1	3.6529	0.1166	0.0000	-3.9193	down
FBgn0028490	CG31705	6749	11988	211.9359	369.7266	0.0000	0.8106	up
FBgn0040211	hgo	312	577	14.9683	27.5236	0.0000	0.8665	up
FBgn0034660	Loxl2	360	784	14.9126	32.0326	0.0000	1.1023	up
FBgn0043783	CG32444	3405	2186	192.3690	121.4100	0.0000	-0.6572	down
FBgn0263780	CG17684	882	446	12.7314	7.2458	0.0000	-1.0000	down
FBgn0042177	Arts	693	388	11.7474	6.5136	0.0000	-0.8530	down
FBgn0063496	GstE4	56	16	6.3265	1.7823	0.0023	-1.7543	down
FBgn0033574	Spn47C	443	938	20.8686	43.8510	0.0000	1.0622	up
FBgn0261041	stj	9287	4538	97.7213	47.2876	0.0000	-1.0511	down
FBgn0040732	CG16926	3315	1007	280.9009	84.6930	0.0000	-1.7359	down
FBgn0014031	Spat	1000	1655	53.6603	86.2653	0.0000	0.7081	up
FBgn0053105	p24-2	11	821	0.2753	58.8633	0.0000	6.0638	up
FBgn0086698	frtz	260	447	4.8155	8.0787	0.0004	0.7610	up
FBgn0040900	CG17777	98	15	22.5953	3.1870	0.0000	-2.6359	down
FBgn0050083	CG30083	19	82	1.7998	7.6993	0.0000	2.0278	up
FBgn0250907	Cht10	142	57	1.2626	0.5191	0.0000	-1.3176	down
FBgn0013812	Dhc93AB	173	40	0.7262	0.1690	0.0000	-2.0994	down
FBgn0052726	CG32726	625	340	810.6744	436.0094	0.0000	-0.8942	down
FBgn0085256	CG34227	933	1889	283.4640	571.0540	0.0000	0.9987	up
FBgn0031389	CG4259	91	39	5.2192	2.4049	0.0079	-1.2165	down
FBgn0037228	CG1092	469	775	17.0026	24.8292	0.0001	0.7051	up
FBgn0030425	Nep6	936	1729	26.4533	49.8263	0.0000	0.8664	up
FBgn0027620	Acf	674	225	7.5344	2.5459	0.0000	-1.5960	down
FBgn0067779	dbr	1105	605	15.4071	8.7170	0.0000	-0.8859	down
FBgn0001187	Hex-C	1125	2555	51.2039	114.1630	0.0000	1.1645	up
FBgn0034471	Obp56e	887	1888	219.1500	465.5611	0.0000	1.0708	up
FBgn0051036	CG31036	254	611	4.7284	11.1741	0.0000	1.2445	up
FBgn0034437	CG10051	1	24	0.0530	0.6673	0.0018	3.5391	up
FBgn0034618	CG9485	783	1430	10.5641	18.9964	0.0000	0.8499	up
FBgn0032076	Argl	801	1590	43.2590	78.4679	0.0000	0.9700	up
FBgn0031824	CG9547	209	382	10.8621	19.4294	0.0001	0.8485	up

FBgn0000047	Act88F	47	109	2.3404	5.2936	0.0034	1.1761	up
FBgn0040364	CG11378	986	570	57.9594	32.8672	0.0000	-0.8075	down
FBgn0035583	CG13704	107	375	16.6566	57.6708	0.0000	1.7803	up
FBgn0029922	CG14431	817	535	8.6031	5.6849	0.0015	-0.6278	down
FBgn0038865	cDIP	366	591	13.3424	21.1755	0.0014	0.6715	up
FBgn0033354	FANCI	90	37	1.3200	0.5425	0.0042	-1.2744	down
FBgn0035266	Gk2	692	1033	22.0868	31.9436	0.0078	0.5591	up
FBgn0015039	Cyp9b2	1455	2970	60.4922	121.8560	0.0000	1.0107	up
FBgn0036757	Ir75a	114	1	3.8236	0.0340	0.0000	-5.7603	down
FBgn0260793	2mit	2658	1832	15.8153	11.1238	0.0022	-0.5548	down
FBgn0040817	CG14132	209	546	19.4022	50.1237	0.0000	1.3625	up
FBgn0038761	CG17190	259	95	25.4314	9.3079	0.0000	-1.4540	down
FBgn0031169	CG1494	191	10	2.3913	0.1239	0.0000	-4.1250	down
FBgn0025583	BomS2	1754	588	1009.1800	344.7760	0.0000	-1.5930	down
FBgn0038820	CG4000	3201	937	288.7060	82.4894	0.0000	-1.7893	down
FBgn0037846	CG6574	3169	2055	124.8072	84.2345	0.0000	-0.6427	down
FBgn0058042		1218	2073	104.8150	179.8760	0.0000	0.7485	up
FBgn0032507	CG9377	443	1130	32.4183	81.6328	0.0000	1.3306	up
FBgn0050145	Obp57e	32	0	13.4574	0.0000	0.0000	-4.8656	down
FBgn0052856	CG32856	72	22	14.8011	5.2278	0.0003	-1.6776	down
FBgn0000477	DNaseII	946	1615	59.6366	100.4110	0.0000	0.7528	up
FBgn0044050	Ilp3	67	12	7.7580	1.3870	0.0000	-2.3914	down
FBgn0010226	GstS1	1705	2513	83.9634	123.4044	0.0040	0.5412	up
FBgn0032921	Mpp6	121	52	12.4185	6.7017	0.0006	-1.2185	down
FBgn0035205	Ctr9	2073	3726	33.1443	59.1263	0.0000	0.8274	up
FBgn0035978	UGP	2287	3972	69.7206	124.3928	0.0000	0.7780	up
FBgn0000055	Adh	50726	87035	3968.3251	6649.1576	0.0000	0.7607	up
FBgn0026565	Ass	131	235	6.9859	12.6788	0.0049	0.8195	up
FBgn0030187	Ipod	23	1	1.1002	0.0549	0.0026	-3.5063	down
FBgn0032322	CG16743	164	390	24.3638	57.1766	0.0000	1.2259	up
FBgn0033302	Cyp6a14	98	37	3.2234	0.8327	0.0004	-1.3958	down
FBgn0052698	CARPB	845	550	14.6393	8.6588	0.0010	-0.6366	down
FBgn0000163	baz	3000	2051	16.4451	13.6339	0.0012	-0.5665	down
FBgn0025885	Inos	10201	16115	384.0000	587.7550	0.0000	0.6415	up
FBgn0023520	CG3857	1041	670	30.3895	19.4896	0.0003	-0.6530	down
FBgn0033926	Arc1	12985	4126	349.8440	110.2140	0.0000	-1.6719	down
FBgn0036783	CheA75a	78	19	10.8042	2.6642	0.0000	-1.9916	down
FBgn0037684	Srr	275	476	13.1245	21.7506	0.0002	0.7709	up
FBgn0083956	CG34120	5389	8220	46.3985	68.6380	0.0003	0.5909	up
FBgn0039348	Npl4	2263	1458	70.7686	44.9403	0.0000	-0.6520	down
FBgn0004959	phm	68	18	2.4270	0.6533	0.0001	-1.8702	down
FBgn0037845	CG14694	186	87	10.6595	4.9435	0.0001	-1.1042	down
FBgn0039848	Loxl1	535	836	25.3005	40.4578	0.0016	0.6248	up
FBgn0035957	CG5144	48	3	2.8597	0.1793	0.0000	-3.5834	down
FBgn0031646	snsl	98	15	7.0529	1.2873	0.0000	-2.6359	down
FBgn0012042	AttA	365	1614	34.5813	159.8007	0.0000	2.1231	up
FBgn0037709	CG8199	180	421	7.1134	16.4820	0.0000	1.2026	up
FBgn0259985	Mppe	2313	3580	97.1341	151.8944	0.0002	0.6118	up
FBgn0265274	Inx3	947	1526	32.7566	52.5599	0.0000	0.6695	up
FBgn0069973	CG40485	2534	4069	129.0802	208.4698	0.0000	0.6649	up
FBgn0086907	Cyt-c-d	30	121	3.2436	12.9288	0.0000	1.9539	up
FBgn0085485	CG34456	8	253	1.7439	47.6702	0.0000	4.7798	up
FBgn0038681	Cyp12a4	1208	1857	50.1880	76.7277	0.0005	0.6018	up
FBgn0040256	Ugt35C1	528	1305	23.5221	57.9883	0.0000	1.2855	up
FBgn0035194	Psf1	55	9	7.7237	1.2521	0.0000	-2.4855	down
FBgn0034132	S-Lap8	34	3	1.2300	0.1067	0.0001	-3.0996	down
FBgn0039104	CG10252	38	0	4.1878	0.0000	0.0000	-5.1057	down
FBgn0058178		3468	2368	81.9398	57.2537	0.0011	-0.5683	down
FBgn0038291	CG3984	54	890	2.8224	45.5040	0.0000	3.9965	up
FBgn0040827	CG13315	2829	1592	484.6960	278.7274	0.0000	-0.8471	down
FBgn0266369	Mtp	1073	1616	21.7714	32.7455	0.0021	0.5721	up
FBgn0000406	Cyt-b5-r	4249	9247	207.3161	447.3381	0.0000	1.1035	up
FBgn0036316	nebu	3177	4731	70.6302	112.0905	0.0016	0.5562	up
FBgn0036992	Hpd	1249	3508	75.5579	207.4062	0.0000	1.4709	up
FBgn0038412	Zip89B	275	528	9.7621	18.4989	0.0000	0.9202	up
FBgn0027571		18066	32839	138.2493	245.5309	0.0000	0.8440	up
FBgn0026721	fat-spondin	2385	3450	69.5743	98.3833	0.0094	0.5143	up
FBgn0051374	sals	1136	770	20.3651	13.6684	0.0032	-0.5785	down
FBgn0035575	CG7509	86	247	2.7762	7.9054	0.0000	1.4917	up
FBgn0037298	Sccpdh1	4940	2976	221.7491	130.5721	0.0000	-0.7490	down
FBgn0030159	CG9689	1139	689	141.6624	70.1960	0.0000	-0.7424	down
FBgn0039678	Obp99a	26	2	6.4803	0.4849	0.0025	-3.1245	down
FBgn0031860	CG11236	120	369	8.1463	24.7841	0.0000	1.5933	up
FBgn0010385	Def	11	82	5.8101	41.4895	0.0000	2.7582	up
FBgn0025454	Cyp6g1	3024	7475	110.6051	268.6622	0.0000	1.2872	up
FBgn0036731	CG6333	43	7	1.9504	0.3068	0.0002	-2.4551	down
FBgn0051469	CG31469	28	185	5.9740	38.6900	0.0000	2.6574	up
FBgn0037057	CG10512	755	1134	30.3734	46.6352	0.0048	0.5680	up
FBgn0032029	CG17292	411	779	18.1024	35.0754	0.0000	0.9025	up
FBgn0036436	CG4914	46	1	1.9886	0.0437	0.0000	-4.4719	down
FBgn0033421	CG1888	464	747	22.4950	33.7354	0.0006	0.6675	up
FBgn0031313	CG5080	1758	2754	58.6657	88.8578	0.0001	0.6291	up
FBgn0030921	CG6290	1002	420	122.4580	50.4705	0.0000	-1.2702	down
FBgn0035206	sturkopf	509	987	26.0068	49.9841	0.0000	0.9357	up
FBgn0040321	GNBP3	1214	1799	57.5531	84.3361	0.0042	0.5489	up
FBgn0032908	CG9270	13	97	0.2065	1.4965	0.0000	2.7774	up
FBgn0025456	CREG	250	452	25.4761	44.7835	0.0000	0.8334	up
FBgn0032287	CG6415	493	749	31.2042	46.6112	0.0082	0.5841	up
FBgn0035282	CNMa	273	59	10.1896	2.4486	0.0000	-2.2064	down
FBgn0036787	CG4306	293	473	23.3760	37.7795	0.0038	0.6707	up
FBgn0261436	DhpD	689	1112	30.4901	48.5559	0.0001	0.6716	up
FBgn0036875	CG9449	408	644	16.9579	26.1337	0.0026	0.6389	up

FBgn0037975	CG3397	317	627	20.0567	39.0562	0.0000	0.9633	up
FBgn0038179	CG9312	475	864	56.4244	101.0860	0.0000	0.8434	up
FBgn0031515	CG9664	610	993	15.2063	24.0085	0.0001	0.6838	up
FBgn0032124	CG17855	48	10	1.8668	0.4012	0.0005	-2.1578	down
FBgn0005626	ple	1305	746	23.8224	13.0060	0.0000	-0.8240	down
FBgn0035147	Gale	1490	2293	70.7135	107.7810	0.0004	0.6034	up
FBgn0001114	Glt	3679	6402	79.4368	136.7531	0.0000	0.7809	up
FBgn0038385	Fbxl7	205	340	4.5519	7.0163	0.0070	0.7087	up
FBgn0034417	CG15117	233	428	6.6783	12.4658	0.0000	0.8560	up
FBgn0036597	CG4962	1455	187	180.9160	22.9123	0.0000	-2.9703	down
FBgn0039312	CG10514	80	207	4.7030	12.0123	0.0000	1.3411	up
FBgn0027521	CG3679	668	995	29.9724	44.1630	0.0094	0.5559	up
FBgn0050409	CR30409	424	756	35.8965	58.7609	0.0000	0.8145	up
FBgn0039817	CG15553	71	186	2.9285	7.7995	0.0000	1.3573	up
FBgn0026314	Ugt35B1	48	704	2.0252	29.0830	0.0000	3.8249	up
FBgn0028513	CG9254	24	2	1.2114	0.1027	0.0081	-3.0140	down
FBgn0013949	Elal	710	1232	52.7954	86.0672	0.0000	0.7760	up
FBgn0028516	ZnT35C	0	43	0.0000	1.8853	0.0000	5.2615	up
FBgn0038290	CG6912	35	2451	1.5448	79.7932	0.0000	6.0663	up
FBgn0023076	Clk	53	216	0.8978	3.9597	0.0000	1.9859	up
FBgn0034491	Hsl	1268	717	24.5923	13.9265	0.0000	-0.8396	down
FBgn0039730	CG7903	1160	780	51.9405	34.7737	0.0020	-0.5900	down
FBgn0039311	CG10513	66	137	4.0205	8.1970	0.0053	1.0229	up
FBgn0261402	Ir75b	36	0	1.3146	0.0000	0.0000	-5.0300	down
FBgn0028473	Non1	6983	10653	213.5580	321.4334	0.0004	0.5911	up
FBgn0038194	Cyp6d5	5468	8135	228.4790	339.8060	0.0018	0.5549	up
FBgn0036259	CG9760	158	79	6.3025	3.1604	0.0020	-1.0076	down
FBgn0038337	CG6125	655	360	23.5266	12.7502	0.0000	-0.8795	down
FBgn0033868	S-Lap7	44	6	1.5779	0.2244	0.0000	-2.6762	down
FBgn0037898	Dtd	35	96	9.0491	24.6405	0.0005	1.4086	up
FBgn0038751	CG4770	0	30	0.0463	1.4584	0.0000	4.7583	up
FBgn0029639	CG14419	1011	633	135.3040	83.0499	0.0001	-0.6926	down
FBgn0015040	Cyp9c1	713	458	27.9507	17.7195	0.0010	-0.6554	down
FBgn0024987	ssx	2008	4383	35.4420	81.8223	0.0000	1.1076	up
FBgn0038799	MFS9	601	927	26.0704	39.0021	0.0022	0.6061	up
FBgn0052282	Drsl4	2416	449	2172.4500	411.0030	0.0000	-2.4429	down
FBgn0032109	CG17005	115	366	3.9009	11.9492	0.0000	1.6424	up
FBgn0031728	Hsp60C	22	1	0.7161	0.0336	0.0048	-3.4452	down
FBgn0036680	Cpr73D	733	326	22.8081	9.6567	0.0000	-1.1842	down
FBgn0036428	Gbs-70E	1676	2539	34.0676	54.6427	0.0008	0.5808	up
FBgn0050026	CG30026	122	229	11.4823	21.4680	0.0015	0.8842	up
FBgn0085736	CG40472	366	199	103.8330	58.4688	0.0000	-0.8934	down
FBgn0051326	CG31326	1189	2152	51.1451	91.6146	0.0000	0.8372	up
FBgn0032387	CG16965	139	282	4.0736	8.2633	0.0000	0.9966	up
FBgn0053696	CNMaR	200	389	4.3328	8.5408	0.0000	0.9377	up
FBgn0027578	Nepl21	1126	2134	28.9744	54.4736	0.0000	0.9035	up
FBgn0001229	Hsp67Bc	195	87	15.6206	6.9576	0.0000	-1.1719	down
FBgn0031110	Obp19b	1141	405	211.2960	73.8451	0.0000	-1.5098	down
FBgn0036116	CG7888	1098	1707	42.2209	66.7482	0.0003	0.6179	up
FBgn0053774	CG33774	29	4	39.4051	5.5292	0.0081	-2.5667	down
FBgn0039315	CG13658	403	115	19.5552	2.8773	0.0000	-1.8170	down
FBgn0032701	CG10341	139	377	4.9257	13.3365	0.0000	1.4140	up
FBgn0014865	Mtk	59	14	148.7220	36.5088	0.0001	-2.0070	down
FBgn0033936	Achl	2023	3108	27.7857	43.0794	0.0003	0.6011	up
FBgn0033051	Strica	97	13	2.4790	0.3088	0.0000	-2.8120	down
FBgn0033395	Cyp4p2	186	24	7.2373	0.9623	0.0000	-2.9135	down
FBgn0027259	Kmn1	116	39	6.6604	3.1325	0.0000	-1.5628	down
FBgn0010401	Os-C	158	43	41.5195	11.0305	0.0000	-1.8679	down
FBgn0038631	CG7695	545	295	77.9096	42.3523	0.0000	-0.9011	down
FBgn0040629	CAH5	574	1145	43.8587	86.6098	0.0000	0.9767	up
FBgn0038525	CG14329	44	6	3.4196	0.4644	0.0000	-2.6762	down
FBgn0040923	CG11368	4969	2975	3680.8840	2148.5310	0.0000	-0.7580	down
FBgn0035344	Cyp4d20	813	1281	30.6042	47.2614	0.0003	0.6371	up
FBgn0005664	Crys	249	112	8.2870	3.5879	0.0000	-1.1626	down
FBgn0051344	CG31344	550	1013	32.0518	56.8115	0.0000	0.8617	up
FBgn0050285	CG30285	295	89	59.3368	18.0734	0.0000	-1.7340	down
FBgn0024293	Spn43Ab	3247	4948	175.4288	261.4623	0.0004	0.5894	up
FBgn0035022	CG11413	24	78	4.0844	12.5542	0.0003	1.6367	up
FBgn0034733	CG4752	436	790	7.2286	12.8193	0.0000	0.8377	up
FBgn0040091	Ugt317A1	136	299	5.4085	12.1807	0.0000	1.1119	up
FBgn0038172	Adgf-D	550	1327	25.9314	61.6228	0.0000	1.2508	up
FBgn0085350	inaF-C	12382	18335	271.4827	407.7817	0.0036	0.5482	up
FBgn0030593	CG9512	4382	6914	138.0280	217.5518	0.0000	0.6397	up
FBgn0262029	d	81	21	1.0166	0.2596	0.0000	-1.9088	down
FBgn0051205	CG31205	365	1793	30.5671	139.7040	0.0000	2.2747	up
FBgn0036910	Cyp305a1	662	1516	30.0883	67.4798	0.0000	1.1759	up
FBgn0038071	Dtg	45	133	1.1969	3.5361	0.0000	1.5217	up
FBgn0029863	CG3823	374	131	20.5303	7.0932	0.0000	-1.5234	down
FBgn0267348	LanB2	302	722	3.3644	7.8859	0.0000	1.2362	up
FBgn0012036	Aldh	9037	13586	328.7940	486.7370	0.0011	0.5700	up
FBgn0020908	Scp1	378	227	30.9765	19.6500	0.0013	-0.7509	down
FBgn0036834	CG6836	251	417	19.6740	31.7801	0.0023	0.7117	up
FBgn0040064	yip2	1808	2851	103.0400	160.1650	0.0001	0.6386	up
FBgn0030575	CG5321	464	719	23.5896	34.5269	0.0040	0.6125	up
FBgn0029990	CG2233	14330	30383	724.1220	1537.7600	0.0000	1.0660	up
FBgn0016684	NaPi-T	27	3	0.7363	0.0819	0.0064	-2.7790	down
FBgn0030482	CG1673	324	665	9.2589	18.8877	0.0000	1.0167	up
FBgn0026056	Rlip	1416	910	36.8913	23.8521	0.0001	-0.6553	down
FBgn0260474	CG30002	317	579	13.0405	24.0249	0.0000	0.8487	up
FBgn0035187	Trh	441	282	13.1536	8.2626	0.0062	-0.6611	down
FBgn0004865	Eip78C	1063	677	22.1801	11.7507	0.0001	-0.6681	down
FBgn0035777	CG8563	6	48	0.2039	1.7370	0.0000	2.7658	up

FBgn0017558	Pdk	2555	4666	53.1850	97.5217	0.0000	0.8504	up
FBgn0037798	CG12817	169	91	11.7831	6.4655	0.0079	-0.9028	down
FBgn0052462	CG32462	48	3	2.9477	0.1826	0.0000	-3.5834	down
FBgn0038654	CG14298	201	362	23.3005	36.4176	0.0002	0.8271	up
FBgn0035484	CG11594	1306	2081	47.6812	74.9664	0.0000	0.6535	up
FBgn0031519	Fign	8	38	0.2406	1.0785	0.0099	2.0812	up
FBgn0038680	Cyp12a5	367	103	14.7647	4.1480	0.0000	-1.8397	down
FBgn0032008	CG14277	3615	2463	607.0240	431.4922	0.0009	-0.5715	down
FBgn0038180	Cht5	50	178	1.6563	5.7562	0.0000	1.7906	up
FBgn0052551	CG32551	83	13	33.4689	5.2612	0.0000	-2.5900	down
FBgn0052191	CG32191	199	585	6.5156	18.7617	0.0000	1.5322	up
FBgn0033289	CG2121	538	1406	20.6502	52.6156	0.0000	1.3659	up
FBgn0033742	Nepl10	143	253	4.5891	8.0769	0.0047	0.8001	up
FBgn0032638	SPH93	318	5	13.1371	0.2128	0.0000	-5.7153	down
FBgn0034443	cer	148	285	43.6779	85.1255	0.0001	0.9220	up
FBgn0003943	Ubi-p63E	5097	8205	126.3493	221.7769	0.0000	0.6686	up
FBgn0000075	amd	319	172	12.6548	6.2304	0.0001	-0.9048	down
FBgn0053207	pxb	248	146	3.0237	1.9422	0.0077	-0.7778	down
FBgn0029828	CG6067	709	1126	38.6157	60.3292	0.0003	0.6484	up
FBgn0035806	PGRP-SD	424	891	53.6725	112.4120	0.0000	1.0512	up
FBgn0040099	lectin-28C	406	1004	8.6740	21.9190	0.0000	1.2857	up
FBgn0027932	Akap200	2759	4097	63.2358	93.4624	0.0020	0.5521	up
FBgn0038912	CG6656	2482	3697	124.5520	185.3810	0.0017	0.5565	up
FBgn0033543	CG12338	894	1330	62.7812	92.4172	0.0055	0.5544	up
FBgn0039759	CG9733	51	153	2.5084	7.2687	0.0000	1.5458	up
FBgn0010383	Cyp18a1	621	1182	14.9604	29.7907	0.0000	0.9092	up
FBgn0001125	Got2	3945	6422	185.7321	294.5470	0.0000	0.6847	up
FBgn0038463	CG3534	439	924	14.7746	30.8333	0.0000	1.0536	up
FBgn0005655	PCNA	18	59	1.3877	4.3236	0.0047	1.6341	up
FBgn0260995	dpr21	989	514	56.8868	30.9927	0.0000	-0.9608	down
FBgn0034885	Eglp4	2961	4537	248.9876	367.5933	0.0003	0.5973	up
FBgn0032782	Rab9	821	1234	37.4777	55.4386	0.0038	0.5691	up
FBgn0050197	CG30197	4306	2850	1219.6900	815.4460	0.0001	-0.6133	down
FBgn0000592	Est-6	4433	6978	166.2002	263.9778	0.0000	0.6363	up
FBgn0037519	CG3014	5540	3242	88.5247	53.5642	0.0000	-0.7909	down
FBgn0036759	CG5577	570	971	30.0182	54.8645	0.0000	0.7492	up
FBgn0005613	Sox15	141	72	2.5126	1.2674	0.0081	-0.9765	down
FBgn0037850	CG14695	60	13	4.6197	0.9915	0.0000	-2.1293	down
FBgn0064237	Idgf5	531	953	26.5298	51.7461	0.0000	0.8243	up
FBgn0035855	CG7366	176	68	3.4967	1.3651	0.0000	-1.3752	down
FBgn0039611	Nepl18	509	1001	16.4813	32.1253	0.0000	0.9560	up
FBgn0041604	dlp	733	1396	11.7752	22.5577	0.0000	0.9102	up
FBgn0025712	CG13920	1011	654	78.9001	51.4371	0.0004	-0.6456	down
FBgn0037624	CG8223	354	148	13.1632	4.6850	0.0000	-1.2697	down
FBgn0038181	CG9297	6129	3778	89.7369	54.9804	0.0000	-0.7160	down
FBgn0038929	CG13408	181	98	11.2272	6.0203	0.0056	-0.8955	down
FBgn0021795	Tapdelta	1028	1732	132.6910	220.0990	0.0000	0.7338	up
FBgn0038589	CG18598	155	341	105.4640	231.6120	0.0000	1.1137	up
FBgn0033287	CG8701	25	0	1.8819	0.0000	0.0001	-4.5232	down
FBgn0051414	Gba1b	345	696	10.5616	21.2493	0.0000	0.9920	up
FBgn0039820	CG15554	9	42	1.0586	4.6343	0.0041	2.0717	up
FBgn0020236	ATPCL	5838	8564	94.2795	136.9460	0.0044	0.5346	up
FBgn0003060	CG9757	24	2	2.2390	0.1805	0.0081	-3.0140	down
FBgn0030362	regucalcin	3786	5738	247.6741	365.5751	0.0005	0.5816	up
FBgn0001225	Hsp26	209	107	16.6017	8.3762	0.0004	-0.9764	down
FBgn0035943	CG5653	405	129	22.7485	7.1534	0.0000	-1.6599	down
FBgn0035791	CG8539	178	60	10.2051	3.1938	0.0000	-1.5687	down
FBgn0002542	lds	50	120	0.9127	2.1611	0.0007	1.2262	up
FBgn0050101	Vajk4	71	170	3.6569	8.7059	0.0000	1.2283	up
FBgn0035160	hng3	168	86	9.3920	4.5393	0.0023	-0.9747	down
FBgn0261575	tobi	834	2621	30.0140	93.0186	0.0000	1.6325	up
FBgn0035770	pst	5204	7862	144.9126	217.6380	0.0007	0.5770	up
FBgn0010039	GstD3	57	184	7.5617	24.1125	0.0000	1.6531	up
FBgn0053143	CG33143	3052	1425	37.7383	14.5568	0.0000	-1.1163	down
FBgn0038214	CG9616	10	87	1.6066	3.3911	0.0000	2.9665	up
FBgn0036765	CG7408	325	191	8.2109	4.6357	0.0014	-0.7814	down
FBgn0037126	CG14567	199	569	26.8682	76.5213	0.0000	1.4922	up
FBgn0038467	AdSL	403	714	11.4173	19.9439	0.0000	0.8053	up
FBgn0039049	CG6726	98	197	4.7481	9.4271	0.0006	0.9810	up
FBgn0038926	CG13409	1108	746	40.3789	26.6176	0.0024	-0.5881	down
FBgn0031529	CG9662	190	331	22.9233	40.3864	0.0015	0.7791	up
FBgn0004045	Yp1	11	54	0.5450	2.4710	0.0001	2.1659	up
FBgn0037683	CG18473	8	427	0.5088	24.8515	0.0000	5.5322	up
FBgn0036337	Adk2	2255	3469	104.6941	168.1273	0.0002	0.6030	up
FBgn0052251	Claspin	283	27	3.1518	0.3029	0.0000	-3.3535	down
FBgn0052823	Sdic3	121	0	3.8169	0.0000	0.0000	-6.7476	down
FBgn0039313	CG11892	333	807	14.7167	30.9515	0.0000	1.2561	up
FBgn0033215	Dgat2	56	145	3.8225	9.6985	0.0000	1.3368	up
FBgn0037105	S1P	321	532	5.8730	9.8820	0.0007	0.7087	up
FBgn0038098	CG7381	439	243	9.8983	5.4709	0.0000	-0.8683	down
FBgn0030968	CG7322	800	1528	78.3658	145.8649	0.0000	0.9145	up
FBgn0030071	CG12661	21	0	0.8932	0.0000	0.0009	-4.2836	down
FBgn0002719	Men	6833	12398	211.7584	375.5319	0.0000	0.8413	up
FBgn0034321	CG14502	50	11	3.1675	1.3642	0.0005	-2.0914	down
FBgn0051207	CG31207	323	555	30.6995	52.9773	0.0001	0.7607	up
FBgn0035239	CG18170	111	12	1.5755	0.1341	0.0000	-3.1101	down
FBgn0039900	Syt7	2777	1688	53.7442	34.4212	0.0000	-0.7359	down
FBgn0035189	CG9119	341	764	21.9205	47.8648	0.0000	1.1430	up
FBgn0039031	Gbp3	780	1242	78.9333	123.5100	0.0002	0.6522	up
FBgn0262003	CG42821	149	51	40.3586	9.3306	0.0000	-1.5436	down
FBgn0036659	CG9701	317	710	11.6063	25.5602	0.0000	1.1424	up
FBgn0035197	CG9130	26	1	0.6689	0.0459	0.0004	-3.6753	down

FBgn0038074	Gnmt	635	2971	53.8011	247.0880	0.0000	2.2060	up
FBgn0014903	CG14630	356	685	12.6950	29.8260	0.0000	0.9239	up
FBgn0031305	Iris	1055	2039	43.6259	84.1758	0.0000	0.9317	up
FBgn0044812	TotC	1001	255	206.4080	52.5780	0.0000	-1.9861	down
FBgn0011281	Obp83a	352	190	39.2227	21.0242	0.0000	-0.9036	down
FBgn0028526	CG15293	3944	5973	230.0297	347.1690	0.0005	0.5805	up
FBgn0039298	to	873	395	56.1001	25.9544	0.0000	-1.1599	down
FBgn0030864	CG8173	136	60	5.7615	2.5653	0.0004	-1.1833	down
FBgn0036255	Atg12	87	178	25.4285	52.5963	0.0009	1.0052	up
FBgn0031873	Gas41	92	177	10.6085	20.4677	0.0049	0.9175	up
FBgn0038301	CG6654	691	1955	21.0712	58.9850	0.0000	1.4808	up
FBgn0028418	Lk	127	57	22.7945	10.1638	0.0010	-1.1579	down
FBgn0030251	CG2145	1836	2871	63.5187	97.1166	0.0001	0.6265	up
FBgn0000109	Aprt	106	276	10.1931	26.4730	0.0000	1.3531	up
FBgn0040398	CG14629	4751	7972	305.8920	499.7630	0.0000	0.7284	up
FBgn0025709	CNT2	64	20	2.3643	0.7351	0.0018	-1.6409	down
FBgn0027783	SMC2	200	111	2.8811	1.6221	0.0055	-0.8609	down
FBgn0035607	CG4835	78	2	1.3684	0.0355	0.0000	-4.6686	down
FBgn0016078	wun	1175	760	51.4612	33.7336	0.0003	-0.6459	down
FBgn0033458	Lime	26	106	0.8429	3.3415	0.0000	1.9632	up
FBgn0259794	sinah	38	6	2.1546	0.3354	0.0008	-2.4704	down
FBgn0058191	CG40191	933	1421	43.4124	61.7245	0.0015	0.5882	up
FBgn0040502	CG8343	20032	31947	2926.5300	4590.3200	0.0000	0.6552	up
FBgn0262717	Skeletor	219	503	5.1950	11.5485	0.0000	1.1773	up
FBgn0051548	CG31548	605	1157	58.0815	109.1810	0.0000	0.9160	up
FBgn0263077	CG43340	10236	15678	131.5099	195.7888	0.0003	0.5969	up
FBgn0003507	srp	1198	1895	36.6555	56.3818	0.0001	0.6429	up
FBgn0051704	CG31704	109	230	29.8370	63.7212	0.0000	1.0514	up
FBgn0033631	Sod3	946	1450	62.9127	110.6190	0.0010	0.5974	up
FBgn0032587	CG5953	650	380	9.8631	6.5869	0.0000	-0.7907	down
FBgn0033911	VGAT	1827	1251	47.0336	32.1087	0.0023	-0.5641	down
FBgn0051973	Cda5	1161	615	13.1873	6.8412	0.0000	-0.9336	down
FBgn0035392	CG1271	287	509	12.1342	20.7411	0.0000	0.8060	up
FBgn0038038	Sccpdh2	905	1871	45.9722	93.4005	0.0000	1.0288	up
FBgn0036790	AstC-R1	268	592	6.4986	14.6135	0.0000	1.1219	up
FBgn0036463	Reck	274	165	4.2703	2.5642	0.0087	-0.7458	down
FBgn0052212	CG32212	26	0	3.9824	0.0000	0.0000	-4.5774	down
FBgn0002565	Lsp2	1455	11203	48.5729	369.5600	0.0000	2.9257	up
FBgn0085484	Pdxk	709	1143	34.4604	55.3265	0.0001	0.6700	up
FBgn0032821	CdGAPr	2153	1312	18.9774	11.4315	0.0000	-0.7322	down
FBgn0038676	CG6026	38	6	0.4466	0.0703	0.0008	-2.4704	down
FBgn0036549	CG10516	492	971	28.2636	54.8455	0.0000	0.9611	up
FBgn0034647	pirk	58	178	3.0678	9.2781	0.0000	1.5809	up
FBgn0052368	CG32368	49	14	25.8182	7.4584	0.0087	-1.7446	down
FBgn0033312	CG8642	50	138	1.9193	5.1544	0.0000	1.4261	up
FBgn0035915	S-Lap1	133	39	4.5591	1.3238	0.0000	-1.7583	down
FBgn0039094	CG10184	196	396	12.5557	24.9830	0.0000	0.9924	up
FBgn0051380	CG31380	1	23	0.1102	1.3476	0.0033	3.4805	up
FBgn0028374	Hug	1358	928	116.6180	78.4497	0.0033	-0.5668	down
FBgn0030558	CG1461	5158	8449	186.0267	298.2958	0.0000	0.6937	up
FBgn0039099	GILT2	202	362	33.3968	59.2135	0.0003	0.8200	up
FBgn0032283	CG7296	1688	1057	157.3180	96.4939	0.0000	-0.6929	down
FBgn0014455	Ahcy	1330	1982	61.9562	88.6599	0.0028	0.5570	up
FBgn0037265	spartin	803	410	26.6609	13.3048	0.0000	-0.9859	down
FBgn0033879	Echs1	1769	2997	146.8059	243.2401	0.0000	0.7421	up
FBgn0037167	CG11425	189	51	17.3761	4.6711	0.0000	-1.8844	down
FBgn0028499	CG7985	605	396	17.3924	11.0798	0.0043	-0.6281	down
FBgn0031490	CG17264	118	13	3.0623	0.3358	0.0000	-3.0918	down
FBgn0040653	Dso1	1020	682	286.7336	199.0589	0.0021	-0.5980	down
FBgn0051769	CG31769	1481	2289	98.3529	153.3720	0.0003	0.6096	up
FBgn0037222	CG14642	6	71	0.3667	3.9465	0.0000	3.3196	up
FBgn0032381	Mal-B1	193	77	7.8064	3.1010	0.0000	-1.3309	down
FBgn0028396	TotA	2534	1454	513.2360	295.9540	0.0000	-0.8190	down
FBgn0034717	CG5819	289	483	5.8780	9.6280	0.0008	0.7206	up
FBgn0039629	CG11842	353	172	26.3290	12.7260	0.0000	-1.0504	down
FBgn0036262	CG6910	2913	6282	141.3196	304.0246	0.0000	1.0903	up
FBgn0001124	Got1	2930	5229	127.4722	222.2448	0.0000	0.8173	up
FBgn0263199	Galk	1968	3054	65.5648	97.5760	0.0002	0.6156	up
FBgn0004654	Pgd	1761	3053	78.7070	132.2511	0.0000	0.7753	up
FBgn0265606	Prosalpha4T1	24	0	2.4938	0.0000	0.0001	-4.4670	down
FBgn0029831	CG5966	1226	729	32.5401	19.4151	0.0000	-0.7672	down
FBgn0026403	Ndg	478	104	6.9843	1.4783	0.0000	-2.2061	down
FBgn0031148	Cbs	1279	1898	51.1176	74.0179	0.0037	0.5509	up
FBgn0033668	exp	248	132	4.9732	2.8051	0.0003	-0.9220	down
FBgn0265416	Neto	442	278	5.0398	3.3039	0.0033	-0.6848	down
FBgn0051407	CG31407	5193	9860	31.6241	59.6250	0.0000	0.9067	up
FBgn0037415	Osi8	65	17	4.3044	1.1306	0.0001	-1.8835	down
FBgn0031913	CG5958	805	1369	48.6577	81.9393	0.0000	0.7471	up
FBgn0001223	Hsp22	315	181	26.2077	15.0434	0.0008	-0.8135	down
FBgn0026602	Adk3	730	1611	19.2991	40.4480	0.0000	1.1226	up
FBgn0040466	Dlip2	221	101	21.2214	9.7741	0.0000	-1.1389	down
FBgn0033233	Kdm4A	282	165	8.8849	5.2605	0.0029	-0.7871	down
FBgn0000489	Pka-C3	2833	1908	52.8505	36.1638	0.0005	-0.5881	down
FBgn0035227	Iml1	3827	1953	51.8212	26.5618	0.0000	-0.9882	down
FBgn0034076	Jhedup	186	71	7.6966	2.9248	0.0000	-1.3931	down
FBgn0058263	MFS17	1065	627	45.4852	26.7534	0.0000	-0.7813	down
FBgn0040207	kat80	1266	588	24.2018	11.6646	0.0000	-1.1230	down
FBgn0264848	vih	392	235	21.6549	13.1272	0.0010	-0.7535	down
FBgn0002930	nec	3270	4766	156.9420	223.2010	0.0058	0.5252	up
FBgn0032698	CG10336	17	60	1.0914	3.6196	0.0016	1.7353	up
FBgn0035434	Drsl5	2365	668	1376.2400	389.9800	0.0000	-1.8403	down
FBgn0010388	Dro	376	53	43.8350	6.3414	0.0000	-2.8182	down

FBgn0050052	Obp49a	3153	2056	337.2180	216.8550	0.0001	-0.6347	down
FBgn0030576	CG15890	311	511	7.4171	12.1312	0.0013	0.6963	up
FBgn0037410	Osi2	100	651	5.1708	32.4709	0.0000	2.6708	up
FBgn0037779	CG12811	214	354	18.0366	29.9086	0.0062	0.7050	up
FBgn0014019	Rh5	16568	10326	1092.1810	667.4544	0.0000	-0.7002	down
FBgn0000045	Act79B	1465	700	80.3457	36.8644	0.0000	-1.0824	down
FBgn0035252	CG7970	487	794	44.9461	70.6957	0.0002	0.6858	up
FBgn0033153	Gadd45	174	329	8.5749	15.9021	0.0001	0.8965	up
FBgn0037230	Nepl11	2571	3908	77.7532	116.7010	0.0005	0.5858	up
FBgn0029521	Or1a	10	49	0.7220	3.4180	0.0005	2.1528	up
FBgn0041194	Prat2	1371	3295	52.6390	125.1996	0.0000	1.2462	up
FBgn0053120	CG33120	240	446	6.5918	12.4116	0.0000	0.8728	up
FBgn0038613	Vha100-4	49	1	1.3115	0.0263	0.0000	-4.5609	down
FBgn0086704	stops	4325	2533	137.9870	81.1854	0.0000	-0.7897	down
FBgn0085195	CG34166	1183	1860	474.3607	804.1418	0.0001	0.6342	up
FBgn0039052	CG6733	0	71	0.0561	4.1502	0.0000	5.9701	up
FBgn0038397	CG10185	33	105	0.4014	1.2439	0.0000	1.6186	up
FBgn0034330	BomS4	46	0	109.1500	0.0000	0.0000	-5.3740	down
FBgn0032864	CG2493	984	1806	42.6534	77.0718	0.0000	0.8572	up
FBgn0024811	Crk	2344	1422	84.5242	58.3586	0.0000	-0.7387	down
FBgn0027500	spd-2	12	50	0.2092	0.8372	0.0018	1.9430	up
FBgn0004181	Ebp	21	1	1.1916	0.0720	0.0089	-3.3815	down
FBgn0034276	Sardh	154	313	3.7816	7.5410	0.0000	0.9998	up
FBgn0034166	CG6472	146	43	7.1916	2.0761	0.0000	-1.7548	down
FBgn0031489	CG17224	789	533	46.0986	30.9140	0.0070	-0.5830	down
FBgn0039800	Npc2g	5313	3267	748.3860	370.7087	0.0000	-0.7195	down
FBgn0029588	CR14798	15	56	1.4292	5.2039	0.0017	1.8063	up
FBgn0034406	Jheh3	532	894	25.0959	41.6553	0.0000	0.7295	up
FBgn0031538	CG3246	1020	2103	57.4110	116.9730	0.0000	1.0249	up
FBgn0038974	CG5377	208	403	13.1743	25.1376	0.0000	0.9323	up
FBgn0015037	Cyp4p1	730	1598	27.3007	59.7745	0.0000	1.1110	up
FBgn0036583	CG13055	132	60	2.8024	1.3196	0.0010	-1.1406	down
FBgn0053926	CG33926	174	2475	19.9944	279.4594	0.0000	3.8034	up
FBgn0034786	CG13531	460	706	5.2276	7.9952	0.0064	0.5987	up
FBgn0034588	CG9394	2407	4395	67.6084	120.0258	0.0000	0.8502	up
FBgn0033372	CG13742	49	3	1.3171	0.0842	0.0000	-3.6125	down
FBgn0038821	CG17267	118	31	6.2029	1.6549	0.0000	-1.9078	down
FBgn0029898	CG14439	1181	1808	44.0734	66.2385	0.0007	0.5958	up
FBgn0033730	Cpr49Ag	330	591	56.4771	100.5320	0.0000	0.8204	up
FBgn0003888	betaTub60D	469	261	12.9204	6.9864	0.0000	-0.8608	down
FBgn0034191	CG6984	83	170	6.4979	13.3198	0.0013	1.0063	up
FBgn0033953	CG12861	22	1	1.4681	0.0668	0.0048	-3.4452	down
FBgn0002570	Mal-A1	270	128	10.7445	4.9827	0.0000	-1.0881	down
FBgn0030439	CG12716	118	17	5.2747	0.9081	0.0000	-2.7326	down
FBgn0053296	CG33296	305	146	11.3060	5.2926	0.0000	-1.0750	down
FBgn0031320	CG5126	245	474	10.6273	20.1414	0.0000	0.9308	up
FBgn0038740	CG4562	559	964	5.5171	9.5537	0.0000	0.7668	up
FBgn0034328	BomBc1	873	1	284.5320	0.3325	0.0000	-8.6848	down
FBgn0041180	Tep4	8495	3111	113.3972	41.0786	0.0000	-1.4670	down
FBgn0039332	alrm	854	2042	34.6729	82.9342	0.0000	1.2384	up
FBgn0039152	Root	997	510	8.0269	4.0921	0.0000	-0.9836	down
FBgn0053080	CG33080	1561	2388	39.4501	58.4688	0.0005	0.5948	up
FBgn0037643	ScsbetaA	7641	11254	264.6441	387.6656	0.0038	0.5404	up
FBgn0004240	DptA	440	159	106.9400	38.5307	0.0000	-1.4799	down
FBgn0037487	thw	97	36	1.3810	0.5351	0.0003	-1.4195	down
FBgn0033052	SCAP	1495	2602	18.3726	31.7406	0.0000	0.7809	up
FBgn0050489	Cyp12d1-p	50	3	1.7878	0.1296	0.0000	-3.6410	down
FBgn0026755	Ugt37B1	32	4	1.3719	0.1694	0.0016	-2.7036	down
FBgn0259896	NimC1	348	677	10.4389	20.8915	0.0000	0.9397	up
FBgn0015527	peng	1172	635	34.6743	16.4295	0.0000	-0.9011	down
FBgn0037488	CG14607	37	0	2.1346	0.0000	0.0000	-5.0684	down
FBgn0040828	CG13306	5	33	1.3190	7.6142	0.0047	2.4583	up
FBgn0030511	CG11158	147	296	7.3970	14.6756	0.0000	0.9861	up
FBgn0038640	CG7706	264	157	6.2217	3.7194	0.0073	-0.7636	down
FBgn0034802	CNBP	1165	158	33.4198	3.7396	0.0000	-2.8914	down
FBgn0037405	CG1077	46	9	1.3488	0.2648	0.0004	-2.2334	down
FBgn0037144	CG7458	65	141	2.1855	4.6087	0.0015	1.0858	up
FBgn0034394	CG15096	2185	3756	94.8129	160.4061	0.0000	0.7631	up
FBgn0050428	CG30428	249	490	5.4484	13.0575	0.0000	0.9553	up
FBgn0034390	CG15093	457	839	28.4771	50.6859	0.0000	0.8567	up
FBgn0031417	CG3597	642	1252	45.6266	87.4264	0.0000	0.9442	up
FBgn0027560	Tps1	11438	17105	315.0030	448.4580	0.0018	0.5624	up
FBgn0029823	Shmt	1824	3651	82.8617	160.8339	0.0000	0.9826	up
FBgn0030001	cyr	276	141	7.2935	3.6200	0.0000	-0.9813	down
FBgn0053978	CG33978	687	428	4.9895	3.2605	0.0003	-0.6993	down
FBgn0053339	CG33339	52	1	3.6132	0.0682	0.0000	-4.6448	down
FBgn0264343	CG43799	2	36	0.2708	3.5607	0.0000	3.5480	up
FBgn0034144	CG5089	21	1	0.8239	0.0396	0.0089	-3.3815	down
FBgn0035982	CG4461	835	364	80.5107	34.6278	0.0000	-1.2134	down
FBgn0051664	CG31664	161	61	5.4317	2.1000	0.0000	-1.4015	down
FBgn0002526	LanA	617	1146	3.2235	5.9163	0.0000	0.8739	up
FBgn0052185	edin	28	0	13.2033	0.0000	0.0000	-4.6800	down
FBgn0031701	TotM	45	11	9.8711	2.4807	0.0044	-1.9430	down
FBgn0027279	l(1)G0196	5207	8057	45.2970	68.7048	0.0001	0.6116	up
FBgn0036687	CG6652	38	5	1.0719	0.1406	0.0002	-2.6878	down
FBgn0000299	Col4a1	3125	4605	32.4506	46.8471	0.0031	0.5411	up
FBgn0027348	bgm	772	521	19.0341	12.7693	0.0071	-0.5844	down
FBgn0052064	S-Lap4	41	3	1.3855	0.1019	0.0000	-3.3617	down
FBgn0052687	CG32687	1843	2940	52.8134	82.5729	0.0000	0.6553	up
FBgn0085452	CG34423	420	790	71.1200	130.2667	0.0000	0.8915	up
FBgn0039114	Lsd-1	2378	5492	124.0390	282.0339	0.0000	1.1891	up
FBgn0028540	CG9008	386	600	12.8125	19.9532	0.0065	0.6168	up

FBgn0031564	CG2816	36	5	9.0100	1.2565	0.0007	-2.6121	down
FBgn0015575	alpha-Est7	3651	6590	126.8040	226.2020	0.0000	0.8337	up
FBgn0086778	nAChRalpha7	4440	3121	43.2067	30.9940	0.0055	-0.5265	down
FBgn0034664	CG4377	115	54	12.9180	6.1682	0.0064	-1.0925	down
FBgn0020385	pug	3405	5536	99.9583	156.4658	0.0000	0.6829	up
FBgn0086691	UK114	694	1039	122.2654	193.5988	0.0067	0.5633	up
FBgn0261714	Cpn	3274	6009	94.6072	170.9010	0.0000	0.8577	up
FBgn0033501	CG12911	21	1	1.0764	0.0507	0.0089	-3.3815	down
FBgn0037548	CG7900	184	433	6.0179	14.1830	0.0000	1.2115	up
Trans_newGene_22		127	255	3.5934	9.0057	0.0001	0.9812	up
Trans_newGene_37		577	1	3.7022	0.0068	0.0000	-8.0884	down
Trans_newGene_79		113	264	1.3008	3.0465	0.0000	1.1979	up
Trans_newGene_85		18	114	0.3992	2.6183	0.0000	2.5710	up
Trans_newGene_87		309	1218	2.3419	9.3711	0.0000	1.9568	up
Trans_newGene_224		101	28	1.6735	0.4803	0.0000	-1.8270	down
Trans_newGene_310		23	0	0.4082	0.0000	0.0002	-4.4084	down
Trans_newGene_313		3	52	0.0495	0.8666	0.0000	3.6654	up
Trans_newGene_378		579	189	4.8005	1.6342	0.0000	-1.6274	down
Trans_newGene_383		173	70	1.9062	0.7912	0.0000	-1.3094	down
Trans_newGene_420		1149	2087	38.8670	70.0514	0.0000	0.8423	up
