# Supplementary material for: Integrative Role of 14-3-3ε in Sleep Regulation
Source: Int J Mol Sci. 2021 Sep 9;22(18):9748. doi: 10.3390/ijms22189748 (PMC8467329; doi:10.3390/ijms22189748)
Supplement: Supplementary file 1 [file ijms-22-09748-s001.zip › Table S3 Neurotransmitter metabolism gene from Go onology analysis of transcriptome 20210722.pdf]

	Gene ID	Mapped IDs	Regulated ZT2	ZT14	Gene Name<br>Gene Symbol	Panther family/subfamily	Panther protein class
**Tyrosine metabolic process**	DROME|FlyBase=FBgn0005626|UniProtKB=P18459	FBgn0005626	-	down	Tyrosine 3-monooxygenase;ple	TYROSINE 3-MONOOXYGENASE (PTHR11473:SF15)	
	DROME|FlyBase=FBgn0001208|UniProtKB=P17276	FBgn0001208	-	up	Protein henna;Hn	PHENYLALANINE-4-HYDROXYLASE (PTHR11473:SF24)	
	DROME|FlyBase=FBgn0040211|UniProtKB=Q9VKJ0	FBgn0040211	up	up	Homogentisate 1,2-dioxygenase;hgo	HOMOGENTISATE 1,2-DIOXYGENASE (PTHR11056:SF0)	oxygenase(PC00177)
	DROME|FlyBase=FBgn0016013|UniProtKB=Q9VZI8	FBgn0016013	up	up	Fumarylacetoacetase;Faa	FUMARYLACETOACETASE (PTHR43069:SF2)	
	DROME|FlyBase=FBgn0036992|UniProtKB=Q9VPF3	FBgn0036992	up	up	4-hydroxyphenylpyruvate dioxygenase;Hpd	4-HYDROXYPHENYLPYRUVATE DIOXYGENASE (PTHR11959:SF1)	oxygenase(PC00177)
**Glutamin family amino acid biosynthetic prcess**	DROME|FlyBase=FBgn0001125|UniProtKB=Q8IPY3	FBgn0001125	-	up	Aspartate aminotransferase;Got2	ASPARTATE AMINOTRANSFERASE, MITOCHONDRIAL (PTHR11879:SF22)	
	DROME|FlyBase=FBgn0001124|UniProtKB=A1ZAA5	FBgn0001124	up	up	Glutamate oxaloacetate transaminase 1, isoform B;Got1	GLUTAMATE OXALOACETATE TRANSAMINASE 1, ISOFORM B (PTHR11879:SF55)	
	DROME|FlyBase=FBgn0026565|UniProtKB=O97069	FBgn0026565	-	up	Argininosuccinate synthase;Ass	ARGININOSUCCINATE SYNTHASE (PTHR11587:SF2)	ligase(PC00142)
	DROME|FlyBase=FBgn0032076|UniProtKB=Q9VLG9	FBgn0032076	-	up	Uncharacterized protein, isoform B;Argl	ARGININOSUCCINATE LYASE (PTHR43814:SF1)	lyase(PC00144)
	DROME|FlyBase=FBgn0038516|UniProtKB=Q9VEJ3	FBgn0038516	-	up	Pyrroline-5-carboxylate reductase;P5cr-2	PYRROLINE-5-CARBOXYLATE REDUCTASE 2 (PTHR11645:SF0)	
**Serine family amino acid metabolic process**	DROME|FlyBase=FBgn0014031|UniProtKB=Q9W3Z3	FBgn0014031	-	up	Serine--pyruvate aminotransferase;Spat	SERINE--PYRUVATE AMINOTRANSFERASE (PTHR21152:SF24)	transaminase(PC00216)
	DROME|FlyBase=FBgn0032287|UniProtKB=Q9VKR4	FBgn0032287	-	up	Aminomethyltransferase;151967_at	AMINOMETHYLTRANSFERASE, MITOCHONDRIAL (PTHR43757:SF2)	methyltransferase(PC00155)
	DROME|FlyBase=FBgn0039094|UniProtKB=Q9VCK6	FBgn0039094	-	up	LD34157p;DmelCG10184	L-THREONINE ALDOLASE (PTHR48097:SF9)	
	DROME|FlyBase=FBgn0029823|UniProtKB=Q9W457	FBgn0029823	up	up	Serine hydroxymethyltransferase;Shmt	SERINE HYDROXYMETHYLTRANSFERASE, CYTOSOLIC (PTHR11680:SF21)	methyltransferase(PC00155)
	DROME|FlyBase=FBgn0031148|UniProtKB=Q9VRD9	FBgn0031148	-	up	Cystathionine beta-synthase;Cbs	CYSTATHIONINE BETA-SYNTHASE (PTHR10314:SF8)	lyase(PC00144)
	DROME|FlyBase=FBgn0037684|UniProtKB=Q9VHF0	FBgn0037684	-	up	LP08712p;Srr	LP08712P (PTHR48078:SF6)	
